# Supra-orbital whiskers act as wind-sensing antennae in rats

**DOI:** 10.1371/journal.pbio.3002168

**Published:** 2023-07-06

**Authors:** Matias Mugnaini, Dhruv Mehrotra, Federico Davoine, Varun Sharma, Ana Rita Mendes, Ben Gerhardt, Miguel Concha-Miranda, Michael Brecht, Ann M. Clemens

**Affiliations:** 1 Neural Systems & Behavior, Marine Biological Laboratory, Woods Hole, Massachusetts, United States of America; 2 Laboratory of Physiology and Algorithms of the Brain, Leloir Institute (IIBBA-CONICET), Buenos Aires, Argentina; 3 Integrated Program in Neuroscience, McGill University, Montréal, Québec, Canada; 4 Montreal Neurological Institute and Hospital, Montréal, Québec, Canada; 5 Instituto de Ingeniería Eléctrica, Facultad de Ingeniería, Universidad de la República, Montevideo, Uruguay; 6 School of Biological Sciences & Graduate Program in Quantitative Biosciences, Georgia Institute of Technology, Atlanta, Georgia, United States of America; 7 Champalimaud Neuroscience Programme; Champalimaud Foundation, Doca de Pedrouços, Lisbon, Portugal; 8 Bernstein Center for Computational Neuroscience, Humboldt University of Berlin, Berlin, Germany; 9 University of Edinburgh, Simons Initiative for the Developing Brain, Edinburgh, Scotland, United Kingdom; Ecole Polytechnique Federale de Lausanne, SWITZERLAND

## Abstract

We know little about mammalian anemotaxis or wind sensing. Recently, however, Hartmann and colleagues showed whisker-based anemotaxis in rats. To investigate how whiskers sense airflow, we first tracked whisker tips in anesthetized rats under low (0.5 m/s) and high (1.5 m/s) airflow. Whisker tips showed increasing movement from low to high airflow conditions, with all whisker tips moving during high airflow. Low airflow conditions—most similar to naturally occurring wind stimuli—engaged whisker tips differentially. Most whiskers moved little, but the long supra-orbital (lSO) whisker showed maximal displacement, followed by the α, β, and A1 whiskers. The lSO whisker differs from other whiskers in its exposed dorsal position, upward bending, length and thin diameter. *Ex vivo* extracted lSO whiskers also showed exceptional airflow displacement, suggesting whisker-intrinsic biomechanics mediate the unique airflow-sensitivity. Micro computed tomography (micro-CT) revealed that the ring-wulst—the follicle structure receiving the most sensitive afferents—was more complete/closed in the lSO, and other wind-sensitive whiskers, than in non-wind-sensitive whiskers, suggesting specialization of the supra-orbital for omni-directional sensing. We localized and targeted the cortical supra-orbital whisker representation in simultaneous Neuropixels recordings with D/E-row whisker barrels. Responses to wind-stimuli were stronger in the supra-orbital whisker representation than in D/E-row barrel cortex. We assessed the behavioral significance of whiskers in an airflow-sensing paradigm. We observed that rats spontaneously turn towards airflow stimuli in complete darkness. Selective trimming of wind-responsive whiskers diminished airflow turning responses more than trimming of non-wind-responsive whiskers. Lidocaine injections targeted to supra-orbital whisker follicles also diminished airflow turning responses compared to control injections. We conclude that supra-orbital whiskers act as wind antennae.

## Abstract 2

Animals rely on sensory processing of airflow (anemotaxis) to guide navigation and survival. This study uses structural, electrophysiological and behavioral analyses to reveal that an under-studied subset of whiskers, the supra-orbital whiskers, act as wind antennae in rats.

## Introduction

Animals can react to airflow stimuli and such wind-sensing abilities are referred to as anemotaxis. The best-studied examples of such behaviors come from insects, where anemotactic turning has been studied, among other species, in crickets [1,2] and in *Drosophila* [3,4]. Crickets show fast [1], highly sensitive [2], and directional escape responses to airflow stimuli. In *Drosophila*, the antennae are important transducers of anemotactic reactions [5]. Until recently, little was known about the anemotactic abilities of mammals, but Hartmann and colleagues showed [6] in a conditioning paradigm that rats can sense airflow. Deficits in airflow sensing after trimming of all whiskers then suggested that this form of airflow sensing is whisker-mediated. The same authors also characterized airflow mechanical responses of mystacial whiskers [7] and responses of rat trigeminal ganglion cells to airflow stimuli [8].

Our work was inspired by the whisker-anemotaxis shown by Hartmann and colleagues. Rather than focus on the 5 rows of mystacial whiskers, which are represented in the famous posteromedial-barrel-subfield [9], we decided to assess the role of all facial whiskers in anemotaxis. The decision to look across different whisker subfields was based on our experience that whisker subfields may have very different functional characteristics. The submandibular whisker trident, for example [10], is a three-whisker array involved in ground sensing. These whiskers appear to possess biomechanical specializations for ground sensing and may provide the animal with ego-motion information about speed and heading direction [10,11]. While the mystacial macrovibrissae have been studied in detail, we know little about the other approximately 300 whiskers on a rat [12]. These whiskers are organized in arrays (the upper and lower lip microvibrissae, the paw whiskers, etc.). The few studies on microvibrissae immediately suggested functional differences between macro- and microvibrissae at the behavioral level [13,14] and the level of cortical representation [15].

The so-called supra-orbital whiskers above the eye are of obvious interest in wind sensing due to their exposed anatomical positioning. Understanding of whisker function comes from understanding how whiskers interact in the environment [16,17]. Our analysis of whisker diversity in wind sensing took advantage of recent progress in automated animal tracking, specifically of the DeepLabCut toolbox [18,19]. We asked the following questions: (i) Which whiskers react maximally to airflow stimuli? (ii) Are whisker airflow responses dependent on whisker biomechanics and substructure? (iii) How do mechanical whisker airflow responses relate to the cortical barrel map? (iv) How do whiskers contribute differentially to airflow sensitivity?

We find that whiskers differ markedly in their airflow responses. In particular, the supra-orbital whiskers respond distinctly when low airflow stimuli are applied, and such airflow responses reflect the specific whisker biomechanics of the supra-orbital whiskers. Micro computed tomography (micro-CT) revealed follicular differences in supra-orbital and pad whiskers. Recordings with Neuropixels probes show increased wind response in the supra-orbital versus pad barrel field. Finally, rats can sense and localize weak airflow stimuli and such abilities are diminished by selective whisker trimming of wind-sensitive whiskers or by blocking supra-orbital whiskers.

## Results

### Differential whisker displacement by airflow

**Fig 1:**
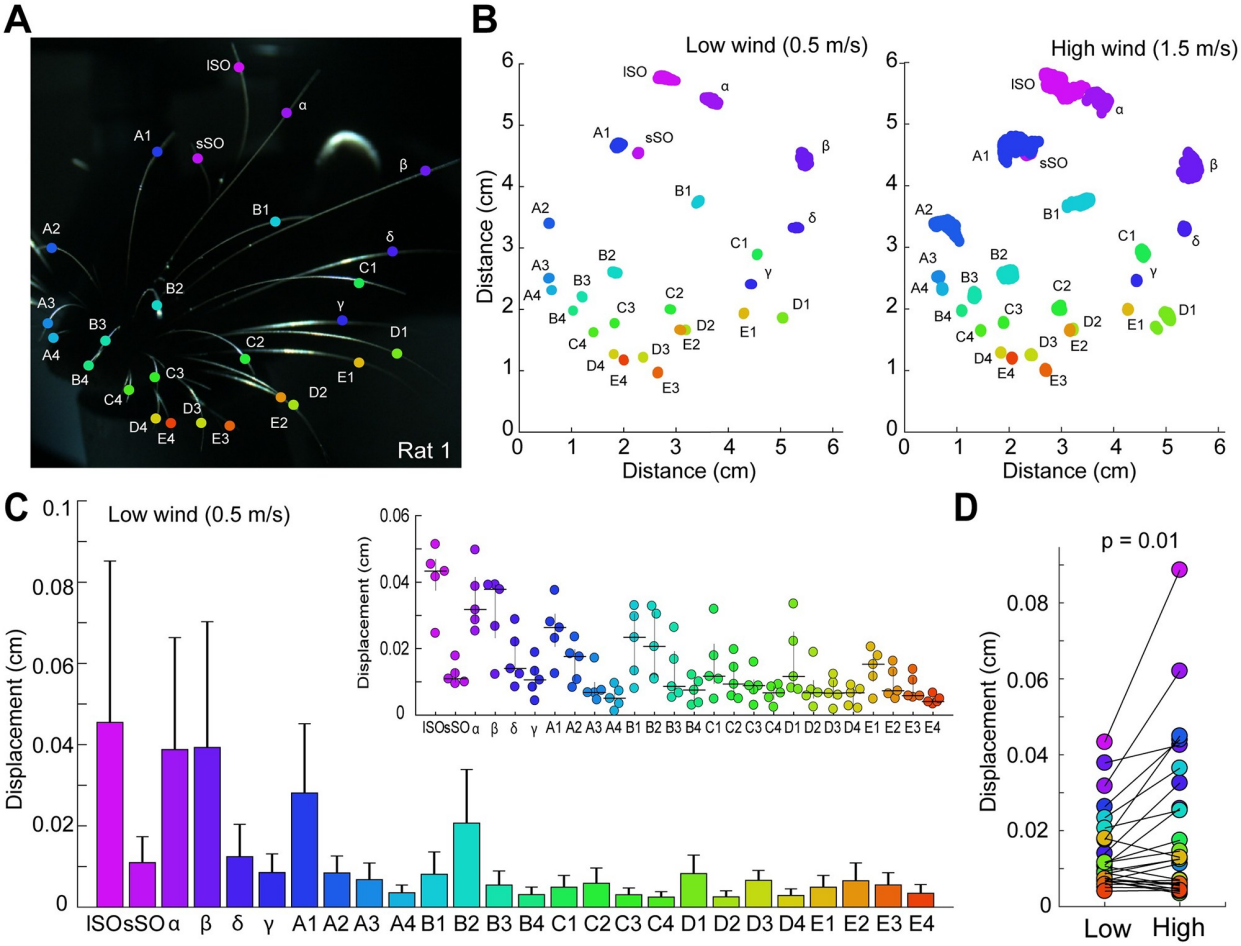
Differential displacement of rat whiskers in regard to airflow. **(A)** Head of a deeply anesthetized rat with whisker tips tracked by DeepLabCut. Whisker tags are color coded and whisker names are indicated. **(B)** Tracked X- and Y-coordinates of the whisker ending segment under low (0.5 m/s; right) and high (1.5 m/s; left) airflow conditions. Note the selective deflection of the lSO, *α*, β, and A1 whiskers. Whiskers are tagged as in A. **(C)** Bar plot depicting whiskers displacement (mean ± SEM), in the same rat as in A. Kruskal–Wallis test: H (25, 166,529) = 93,728.2, *p* < 0.0001. Top right: inset showing whiskers displacement in 5 rats (median ± IQR). Note that lSO whiskers show the largest displacement. **(D)** Scatter plot showing the paired displacement (cm) of whiskers averaged across rats in the low and high wind conditions. Right-tailed Wilcoxon test: *p*-value = 0.01. All data underlying the figure can be accessed through https://figshare.com/s/6a9f2304f6ca45b8a7f8. See also S1–S4 Movies. IQR, interquartile range; lSO, long supra-orbital.

As a first step of our analysis, we assessed the passive displacement of whiskers by wind stimuli, using filmed heads of 5 animals, deeply anesthetized with ketamine, under 2 wind conditions. Whiskers’ end segment was tracked using DeepLabCut [19] (see also S1 and S2 Movies). We labeled the long and short supra-orbital whiskers (lSO and sSO), the straddlers and the whiskers in Arcs 1 to 4 (Fig 1A). We recorded videos of rats under low (0.5 m/s) and high (1.5 m/s) wind conditions. Fan generated turbulent airflow surpassed 80% of its total strength shortly after the stimulus onset and then rapidly reached a steady state [20] (S1A–S1D Fig; S3 and S4 Movies). We then examined the x- and y- movement of each whisker type and calculated displacement as the positive difference between the whisker end segment positions in the 2D plane and their median. We found that while a large number of whiskers exhibited substantial displacement in the high wind condition (Fig 1B), in the low wind condition, only specific whiskers showed marked displacement; these were predominantly the lSO, α, β, A1 whiskers (Fig 1C and S2 Fig).

When grouping the averaged whisker displacement on each rat by whisker type, we observed that the lSO whiskers displayed the highest displacement (Fig 1C, inset). To study this pattern quantitatively, we analyzed rats individually. Kruskal–Wallis and post hoc analyses revealed that lSO whiskers moved more than any other whisker apart from α, β, and A1 whiskers. Among this whisker subset, the lSO moved more than the others in most cases (Fig 1C and S2 Fig). Only in Rats 2 and 4, another whisker (α) displaced significantly more than the lSO.

Next, we characterized the whisker differential movement upon low (0.5 m/s) and high (1.5 m/s) airflow by taking the median of whisker displacement across rats and comparing their paired values on each condition (Fig 1D). Wilcoxon test indicated that there was a significant increase in the whisker displacement in the high wind condition. A more detailed analysis indicated that this effect was due to a change exhibited by nearly a dozen whiskers, most of them belonging to the supra-orbital region and the top, ocular corner of the whisker pad (S3 Fig). While the details of whisker displacements differed across video sequences, two aspects were the same: (i) lSO, α, β, and A1, whiskers as well as some closely neighboring whiskers always showed big displacements; and (ii) anterior and middle whiskers of the C, D, and E rows always showed little airflow induced displacements. In addition to the quantitatively analyzed movies shown in Fig 1, we also inspected a variety of additional rat head movies qualitatively. These movies included videos of head side views and movies of upside-down heads. All of these recordings led to similar qualitative conclusions. Notably, in all of our experiments, the lSO showed very strong and usually the maximal deflection, prompting us to further examine the function of the lSO in detail with regards to anemotaxis in rats.

### Differential whisker biomechanics determine airflow responses

**Fig 2:**
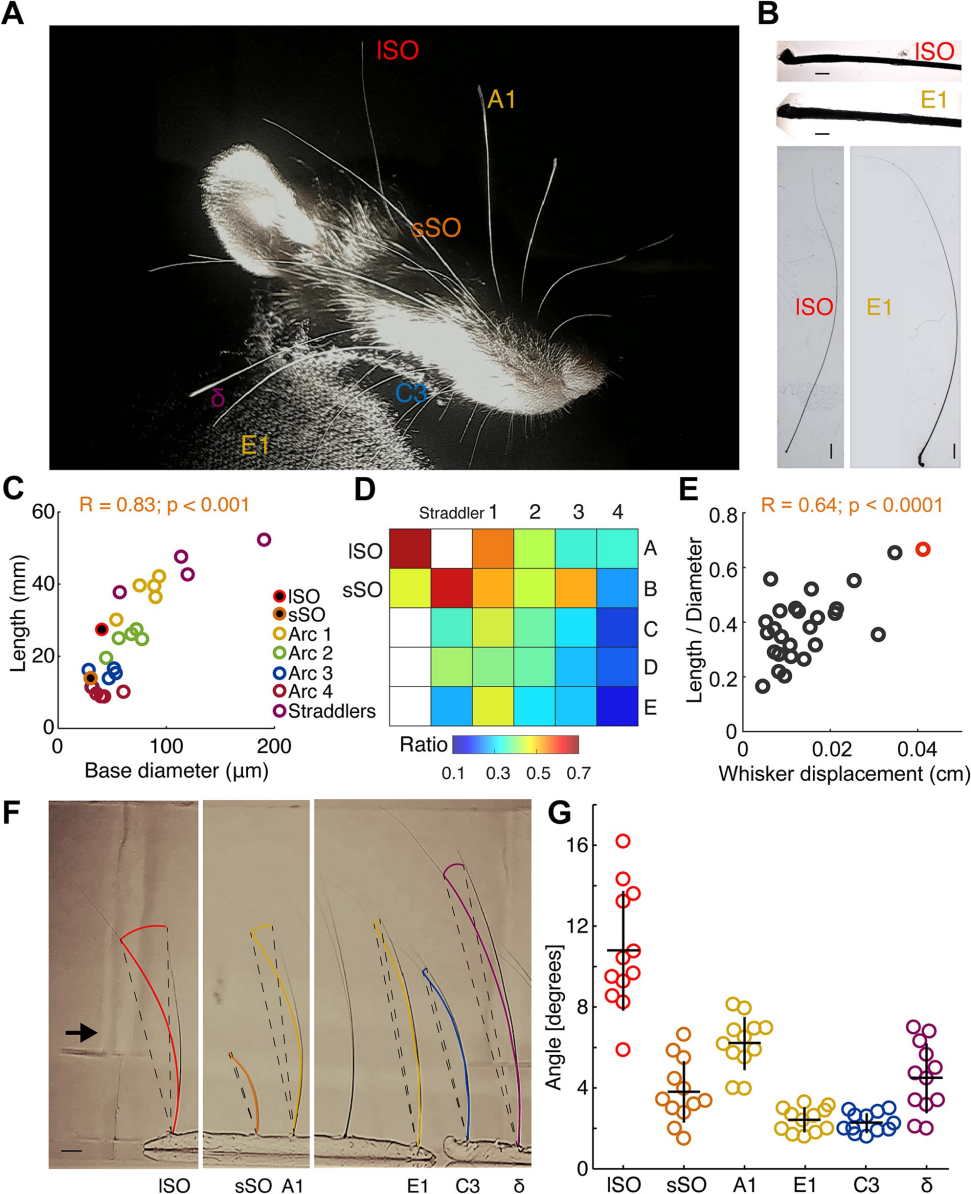
Differential biomechanics determine rat whisker airflow responses. **(A)** Head of a deeply anesthetized rat. Note the thin whisker diameter of the lSO whisker. **(B)** Micrograph of the initial segments of lSO and E1 whiskers (top). Photograph of lSO and E1 whiskers (bottom). Scale: 1 mm. Scale: 100 μm. **(C)** Whisker length plotted against whisker base diameter. Color coded by arcs, inside which length varies the least. Each data point represents the mean length or diameter of each whisker type (*n* = 4). Pearson correlation indicated. **(D)** Heatmap of the ratio between whisker length and base diameter. Note that lSO has the highest ratio (see S1 Fig). **(E)** Pearson correlation between the whisker length to base diameter ratio in regards to whisker displacement. lSO whiskers indicated in red. Rho and *p*-value indicated. **(F)** Whisker bending while blowing wind onto extracted whiskers *ex vivo*. Color-coded curves fitting 75% of the total whisker length were employed to trace a radius (dashed lines) centered at the base of the whisker to calculate the bending angle. Maximal lSO bending is shown. Approximate wind direction (black arrow). Scale: 2 mm (black line, bottom left). **(G)** Bending angle for each whisker type (color coded). Each dot represents the deflection that a given whisker reached when itself or other whisker type reached its maximal bending. Kruskal–Wallis test on whisker type [H (5, 42) = 36.45, *p* < 0.0001]. Dunn’s post hoc test indicated that the lSO bending angle significantly differed from every other whisker (all ps < 0.02) except from A1. Meanwhile, A1 differed from C3 and E1 (ps < 0.01). Black crosses indicate the mean ± SEM. All data underlying the figure can be accessed through https://figshare.com/s/3601b5d34cecd4df8f25. See also S5 Movie. lSO, long supra-orbital.

We wondered how the differential responses of whiskers to airflow arise. To address this question, we first visually inspected whiskers with differing airflow responses. Differential characteristics were readily visible and immediately noted, namely that the lSO whisker was unusually thin for its length (Fig 2A). Such differences were confirmed when we acquired micrographs of full whiskers and their shafts (Fig 2B). Total whisker length and base diameter were measured in wind and non-wind-engaged whiskers (Fig 2C). We computed a Pearson correlation to examine the relationship between whisker length and base diameter and found a positive correlation between the two variables [r (26) = 0.83, *p* < 0.001] (Fig 2C). lSO whiskers were relatively thin and short among the long whiskers (Arc 1, 2, and the straddlers) and display a clear difference with respect to the small supra-orbital and the shorter whiskers (Arc 3 and 4). We computed a heatmap of the ratio between whisker length and base diameter and found that lSO has the highest ratio (Fig 2D). We grouped the different whisker types according to a semicircular arrangement and compared their fold change for that ratio with respect to the lSO whisker. Semicircles were found to minimize the mean variance of the ratio along the whisker pad when compared to other possible arrangements using shuffling statistics. Further statistical analysis confirmed that lSO exhibits the highest ratio (S4A Fig). This result suggests that optimal wind engaging occurs within a length-base diameter range that includes supra-orbital and top semicircle whiskers. To further explore this possibility, we performed Pearson correlations of whisker length, base diameter, and their ratio, against the whiskers displacement under low wind (0.5 m/s; Fig 2E and S3B Fig). Results indicated that only the whisker length and the ratio exhibited a significant correlation with displacement (r = 0.4, *p* = 0.04 and r = 0.64, *p* < 0.0001, respectively). Up to this point, our data on whisker biomechanics shows that in spite of the fact that the lSO whisker has a moderate length (27 mm; distribution median = 24.7 mm and mean = 25mm), it displays the highest proportion between length-base diameter ratio and wind-induced displacement. These results are in line with previous work showing that whiskers with larger ratios (α >A2 >E2 >C2 >D5), which are not necessarily the longest or thinnest, have higher vibration magnitudes at a given wind speed [8].

In order to further evaluate if these biomechanical properties are sufficient to determine differential airflow responses, we performed *ex vivo* experiments on extracted whiskers (Fig 2F). To this end, we inserted the base of a similar sample of wind and non-wind-engaged whiskers in clay on a linear array with similar orientation. We calculated the maximal bending of the whiskers during low wind flow with respect to the curvature at rest and took the bending angle (Fig 2F and 2G; see Materials and methods). A Kruskal–Wallis test on whisker type showed a significant effect [H (5, 42) = 36.45, *p* < 0.0001]. Dunn’s post hoc test indicated that only comparisons involving lSO and A1 whiskers yielded significant differences. Particularly, the bending angle of lSO significantly differs from every other whisker (all *p*-values <0.02) except A1, which was another wind-sensitive whisker found in our previous in vivo assay. A1 differed from C3 and E1 (*p*-values <0.01). Taken together, our results identify whisker biomechanics as crucial determinants of airflow responses.

### The follicles of wind-sensitive whiskers have an unusually closed ring-wulst

**Fig 3:**
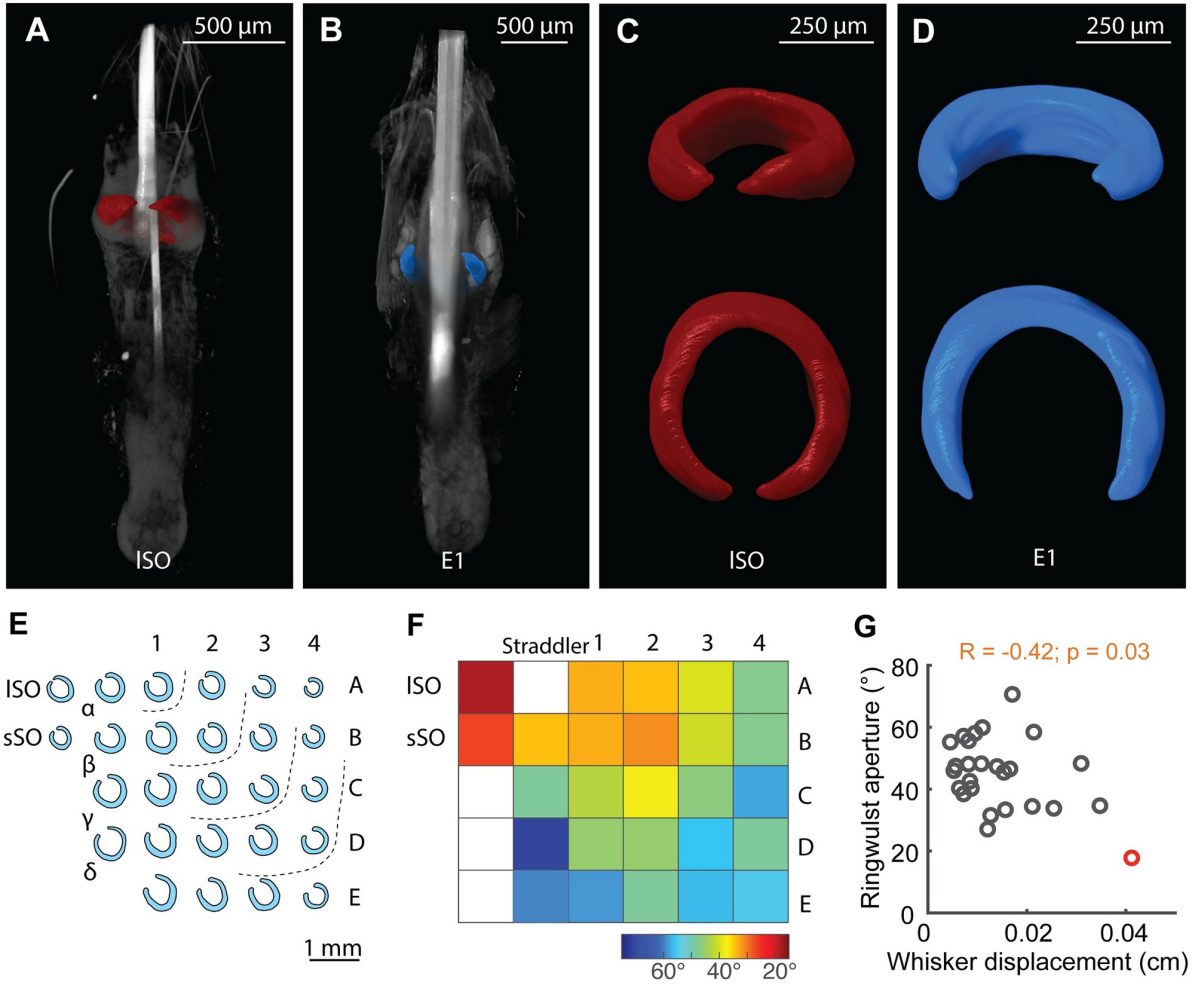
Supra-orbital whiskers and other wind-sensitive whisker have more closed/complete ring-wulst than non-wind-sensitive whiskers. **(A)** Micro-CT scan volume rendering of an lSO vibrissa follicle. Gross anatomy is visualized in gray and ring-wulst reconstructions in color (red). **(B)** As (A) but for the E1 vibrissae follicle (blue ring-wulst). **(C)** Reconstructed lSO ring-wulst from (A) in an oblique and top view. **(D)** Same as (C) but for the E1 follicle from (B). Note the marked difference in the ring-wulst aperture angle between the wind-sensitive lSO and non-wind-sensitive E1 vibrissa. **(E)** Illustration of vibrissa ring-wulst shapes drawn from micro-CT scans. Dotted lines indicate a semicircle like arrangement of vibrissae by ring-wulst aperture angles. **(F)** Heatmap of ring-wulst aperture angles. Measurements were taken from the center of the (new) hair shaft to the most distal extension of the ring-wulst in the plane of maximum aperture (*n* = 5). Color bar indicates closed (red) to rather open (blue) conformations. **(G)** Pearson correlation between ring-wulst aperture and whisker displacement. lSO whisker in red. Rho and *p*-value indicated. All data underlying the figure can be accessed through https://figshare.com/s/3601b5d34cecd4df8f25. lSO, long supra-orbital; micro-CT, micro computed tomography.

We next compared the follicle structure of wind-sensitive and non-wind-sensitive whiskers. We obtained high-resolution micro-CT scans of whisker follicles either in situ in entire iodine-stained whisker pads or in extracted single iodine-stained follicles. Our analysis was informed by the seminal work of Tonomura and colleagues [21]. These authors identified structure–function relationships in vibrissa follicles and showed that afferents with club-like endings, which are exclusively found adjacent to the ring-wulst, are the most sensitive follicle afferents with the highest discharge rates. We reckon that such ring-wulst afferents are most likely to respond to wind stimuli, which do not even evoke a visible deflection in many whiskers. We show a volume rendering of the lSO whisker follicle, a highly wind-sensitive whisker in Fig 3A and of the E1 whisker follicle, a non-wind-sensitive whisker in Fig 3B. These two whiskers differ in their ring-wulst, which we reconstructed via manual segmentation, high-lighted by color in the volume image and which we show in isolation in Fig 3C. Wind-sensitive whiskers have a relatively closed ring-wulst (Fig 3C), whereas non-wind-sensitive whiskers tend to have an open ring-wulst (Fig 3D). Population data on ring-wulst opening are plotted in Fig 3E and 3F. Note the similarity of “ring-wulst-closedness” (Fig 3E) and wind-induced deflection as shown in Fig 1. A heatmap of ring-wulst aperture angles indicates the most closed aperture in lSO and sSO follicles, while the most open aperture conformations are found in E-row and arc-4 follicles (Fig 3F). We grouped the different whisker types according to a semicircular arrangement and compared their fold change for the ring-wulst aperture with respect to the lSO whisker. Semicircles were found again to minimize the mean variance when compared to other possible arrangements using shuffling statistics, but this time for the ring-wulst aperture. Further statistical analysis confirmed that lSO exhibits the most closed ring-wulst (S5A Fig). Interestingly, we found that the ratio between whisker length and base diameter was inversely correlated with the ring-wulst aperture: the more closed the ring-wulst, the higher the ratio (r = −0.66; *p* < 0.001; S5B Fig). Ring-wulst aperture also showed a correlation with whisker base diameter (r = 0.44; *p* = 0.02; S5B Fig). Furthermore, ring-wulst aperture did correlate with wind-induced whisker displacement, with lSO whisker displaying the most extreme relation between these variables: the closest ring-wulst and the largest displacement (r = −0.42; *p* = 0.03; Fig 3G). We conclude that the follicles of wind-sensitive whiskers differ by an unusually closed ring-wulst from non-wind-sensitive whiskers.

### Mapping of supra-orbital whisker barrels and relation of whisker airflow displacement to the cortical barrel map

**Fig 4:**
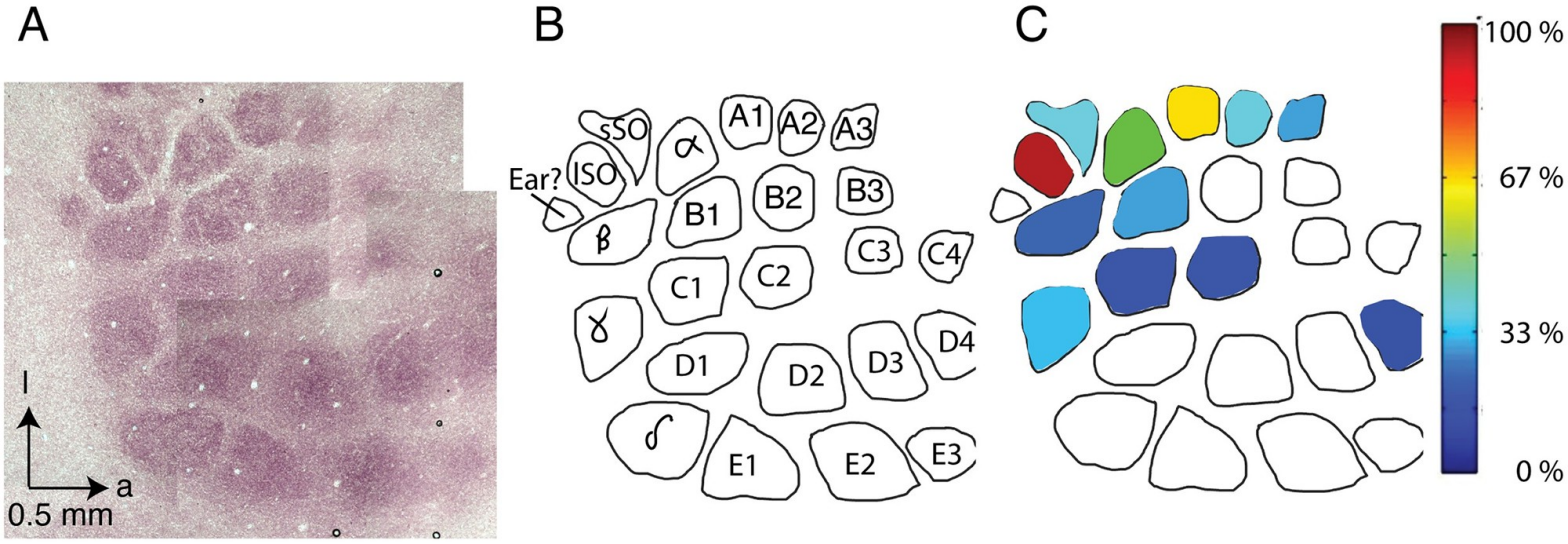
Localization of supra-orbital whisker barrels and relation of whisker airflow displacement to the cortical barrel map. **(A)** Cortical barrels in a tangential section through layer 4 of rat barrel cortex revealed staining for cytochrome oxidase reactivity; dark brown color indicates high reactivity. a = anterior, l = lateral. **(B)** Drawing of cortical barrels (from A) with the positions of supra-orbital whisker barrels. sSO and lSO whisker barrels were identified in 4 receptive field mapping experiments, in all cases posterior rather than lateral to α/A1 whisker responses. Note that some anterior barrels (A4 and B4) and microvibrissae barrels are missing due to sectioning. **(C)** Whisker displacement under low airflow conditions was quantified, normalized to the maximal response, color coded and superimposed to the barrel map drawn in (B). Data comes from an airflow whisker displacement experiment on the head of an anesthetized animal analogous to the data shown in Fig 1F. Quantitative tracking data for whisker displacement were not available for all whiskers (hence, the empty barrels). Qualitative assessment of D- and E-row whiskers suggested they show little airflow whisker displacement similar to the data of whisker D4 (also see S1 Movie). Wind-responsive whiskers (with large airflow displacements) cluster in the posterolateral barrel map. All data underlying the figure can be accessed through https://figshare.com/s/e563353889ea06181807. lSO, long supra-orbital; sSO, short supra-orbital.

The differential mechanical airflow responses of whiskers point towards a role of the supra-orbital whiskers in airflow sensing. We therefore mapped the location of cortical barrels representing the supra-orbital whiskers in extracellular receptive field mapping experiments and prepared cytochrome oxidase sections of layer IV of the barrel cortex (Fig 4A) [22]. We consistently (in 4 out of 4 mapping experiments) observed supra-orbital whisker responses in brain regions posterior to the A1 and α whisker response areas. Also, the stereotaxic coordinates of supra-orbital whiskers were highly consistent (6.26 ± 0.01 mm lateral and 3.75 ± 0.20 mm posterior to bregma, mean ± SEM). These observations led us to a suggestion for the location of the supra-orbital whisker barrels in relation to the rest of the barrel field (Fig 4B). Next, we wondered how mechanical airflow responsiveness relates to the cortical barrel field and we color coded it and superimposed it on the barrel map (Fig 4C). Quantitative tracking data for whisker displacement was not available for all whiskers (hence, the empty barrels), but it was nonetheless clear that wind-responsive whiskers (with large airflow displacements) cluster in the posterolateral barrel map.

We also inspected the putative supra-orbital whisker barrels in many (*n* = 10) additional barrel maps that we obtained from other purposes in previous studies [23,24]. We made the following observations: (i) the exact position and orientation of putative supra-orbital whisker barrels relative to the posteromedial-barrel-subfield is somewhat variable and more variable relative to the position and orientation of the mystacial barrels to each other. (ii) Putative supra-orbital whisker barrels are elongated. (iii) Putative supra-orbital whisker barrels are always close (see also Fig 4A and 4B). (iv) The septum separating putative supra-orbital whisker barrels is weaker than the septum separating mystacial barrels (see also Fig 4A and 4B). The latter 2 observations support the idea that the short and long supra-orbital whiskers are functionally related.

### Neurons in the supra-orbital whisker representation respond more strongly to wind stimuli than E/D-row barrel cortex neurons

**Fig 5:**
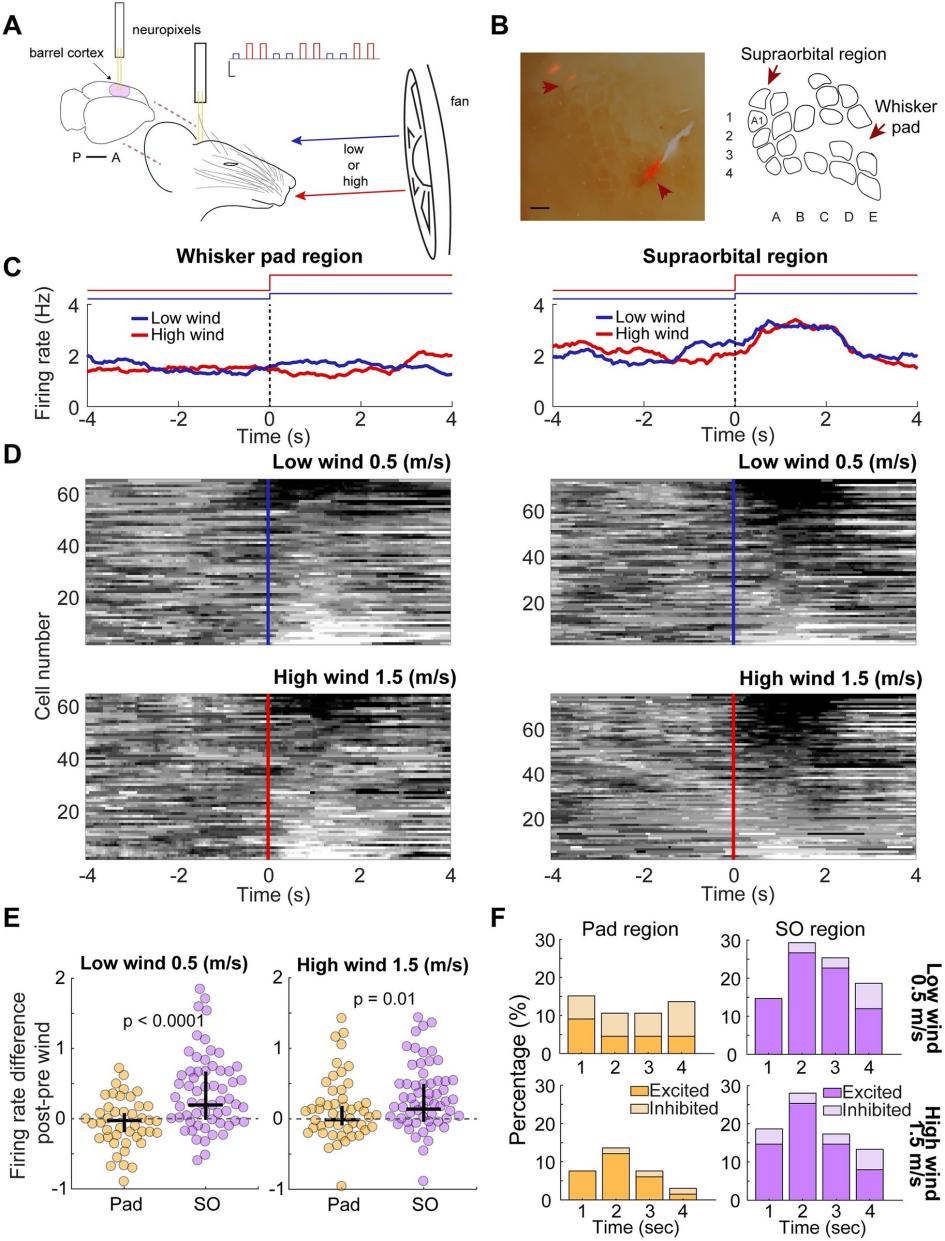
Supra-orbital whisker cortex responds more strongly to wind stimuli than D/E-row barrel cortex. **(A)** Schematic of the experimental setup. Posteriorly and anteriorly placed Neuropixels probes were aimed to the supra-orbital and the whisker pad regions of the barrel cortex, respectively. Simultaneous, contralateral recordings of single units were made while blowing wind. Low (0.5 m/s, blue) or high (1.5 m/s, red) wind epochs (10 s) were blown in alternating order from a frontal fan placed 12.5 cm apart from the rat’s head. Top right: schematic of the wind epochs in time (12–30 total wind epochs per rat). Scales: x: 10 s; y: 1.5 m/s. **(B)** Left: representative histology showing the 2 recording sites on the whisker pad and supra-orbital regions of the barrel cortex. Scale: 500 μm. Right: schematic reconstruction of the barrel cortex from successive flattened brain slices. **(C)** Representative examples of peri-wind stimulus firing rate of 2 single units recorded at the whisker pad (left) or supra-orbital (right) regions in the low (blue) and high (red) wind conditions. Black dash lines and color code step lines on top indicate stimuli onset. (**D)** Heatmap of z-scored firing rate around wind stimuli (low wind, top; high wind, bottom) of single units recorded at the whisker pad (left) or supra-orbital (right) regions. Positive z-scores indicate excitation (black). Negative z-scores indicate inhibition (white). **(E)** Firing rate for the difference between post vs. pre-wind stimulation in single units recorded at the whiskers pad (yellow) or the supra-orbital (lilac) regions for low (left) and high (right) wind conditions. **(F)** Percentages of excited and inhibited pad (left) and supra-orbital (right) regions across time after low (top row) and high (bottom row) wind conditions, calculated using a generalized linear model (GLM). Note that response percentage peaks at second 2. All data underlying the figure can be accessed through https://figshare.com/s/ef2e783e590dc552cf08.

Next, we wondered if the cortical supra-orbital whisker representation differed from barrel cortex neurons in their responses to wind stimuli. We applied wind stimuli to urethane-anesthetized rats, while recording simultaneously with Neuropixel probes from the supra-orbital whisker region at the coordinates identified in our mapping experiments and from the whisker pad region aiming towards E/D-row barrel cortex (Fig 5A). We histologically confirmed recording locations to the supra-orbital cortical region and the whisker pad barrel cortex near E/D-row (Fig 5B). Judging by the population peristimulus time histogram (PSTH), there was not much of a wind-evoked response in recordings from E/D-row barrel cortex. In contrast, there was a clear excitatory response in the supra-orbital whisker region (Fig 5C). Plots of the z-scored responses of individual neurons revealed either no, weak, or inhibitory responses to wind stimuli in E/D-row barrel cortex. In the supra-orbital whisker region instead, we observed strong excitatory responses in single cells (Fig 5D). The differences in the firing rate response to either low (0.5 m/s) or high (1.5 m/s) wind between these cortical regions were highly significant (Fig 5E) and were distributed differently in time across response categories (Fig 5F). We found that the SO region exhibited the highest percentage of excited neurons, surpassing the 25% of recruitment 1 second after the stimulus onset in both wind conditions. In contrast, pad region neurons displayed a balance between being excited and inhibited during low wind and only recruited 12% of neurons at its peak during high wind. This pattern of response was further explained by an analysis on the response latency, which showed that neurons reached their maximum response 1 second after the stimulus onset (S6A Fig). These differences suggest that wind responses map to the supra-orbital whisker barrel. To further confirm this, we calculated for each cell the mutual information of the firing rate given a wind stimulus (S6B Fig). Results indicated that only firing rate activity of neurons in the SO region significantly informed about wind stimuli. Moreover, in line with our results regarding the percentage of recruited activity and the response latency, mutual information peaked during second 2 (low wind, *p* = 0.0007; high wind, *p* < 0.0001).

### Anemotaxic turning in rats

**Fig 6:**
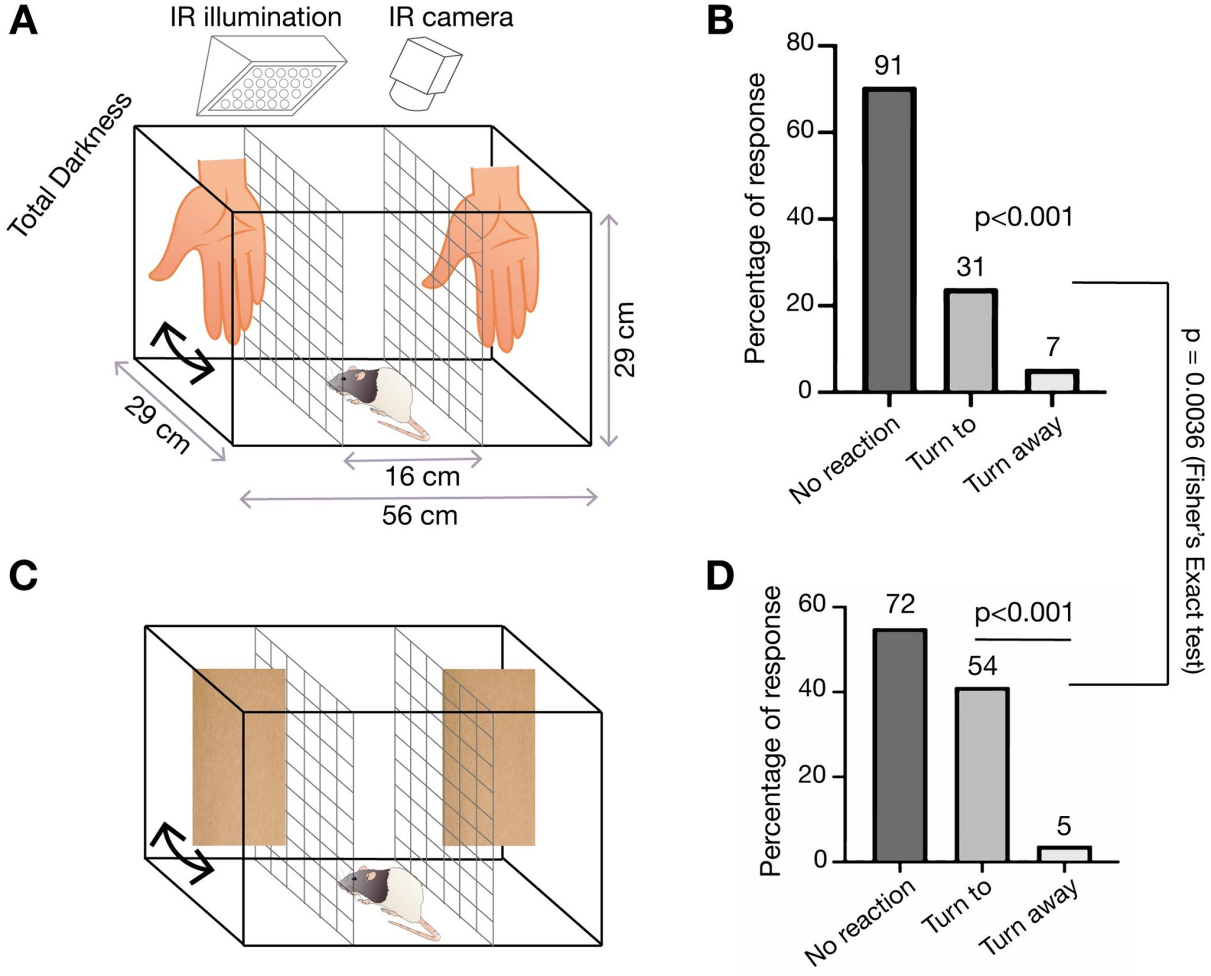
Anemotaxic turning in rats. **(A)** Schematic of the behavioral arena for wind-sensing behavior in response to hand-flapping. See Materials and methods for more details. **(B)** Behavioral responses of rats (*n* = 7) to hand-movement stimuli (0.5 s post stimulus) were assigned by forced choice to one of 3 categories: either no reaction, turning towards the stimulus, or turning away from the stimulus. Rats were strongly biased to turn towards the hand-movement stimuli (*p* < 0.001, χ^2^ test). **(C)** Cardboard-flaps are used to apply stronger airflow stimuli than the hand-flaps. **(D)** Rats react to the cardboard-flap movement stimuli from (C) and scoring is done as in (B). Rats were strongly biased to turn towards the cardboard-flap stimuli (*p* < 0.001, χ^2^ test). Also, rats turn towards cardboard-flaps more frequently than to hand-flaps (*p* = 0.0036, Fisher’s exact test). All data underlying the figure can be accessed through https://figshare.com/s/07af3c1099b5b785acff.

To assess the behavioral capacities for wind sensing in rats, we developed an airflow-sensing paradigm. We placed a rat in a box with 3 compartments separated by wire-mesh in total darkness. The rat was placed in the middle compartment and 2 experimenters performed repetitive hand-flaps or cardboard-flaps, in either one of the 2 lateral compartments (Fig 6A and 6C). Airflow measurements of hand- and cardboard-flap stimuli were on average ≤0.3 m/s and 0.5 m/s, respectively. The reactions of rats to hand-flap stimuli were assigned by forced choice to one of 3 categories: either no reaction, turning towards the stimulus, or turning away from the stimulus (Fig 6B). Even though rats often showed no reaction, when they did, the animals appeared to be able to distinguish the side where the hand-flap was delivered. Accordingly, rats turned significantly more often towards hand-flaps than away from them (Fig 6B; *p* < 0.001, χ^2^ Test; “Turn to” (31 trials) versus “Turn away” (7 trials)). Next, using the same behavioral paradigm, we changed the wind delivery method to utilize a cardboard piece, which induced stronger airflow than the hand-flap (Fig 6C). Again, the animals consistently showed a higher percentage of responses towards the stimuli side when compared to turning away responses (Fig 6D; *p* < 0.001, χ^2^ test). When comparing the “Turn to” responses in the 2 wind delivery methods, we observed a stronger reactivity of the animals to cardboard-flap than to hand-flap stimuli (Fig 6C and 6D; *p* = 0.0036, Fisher’s exact test). Our results show that rats can not only sense, but also turn towards airflow stimuli. The strength of the reactions differed between weak (hand-flap) and strong (cardboard-flap) stimuli. Since we carefully avoided noises associated with hand-flap or cardboard-flap stimuli and conducted experiments in total darkness, it is likely that animals indeed sensed airflow. The whisker trimming and lidocaine injection effects described below show that the turning responses observed were indeed at least partially, if not entirely, tactile reactions.

### Wind-whisker trimming and supra-orbital whisker blockade interfere with airflow turning responses

**Fig 7:**
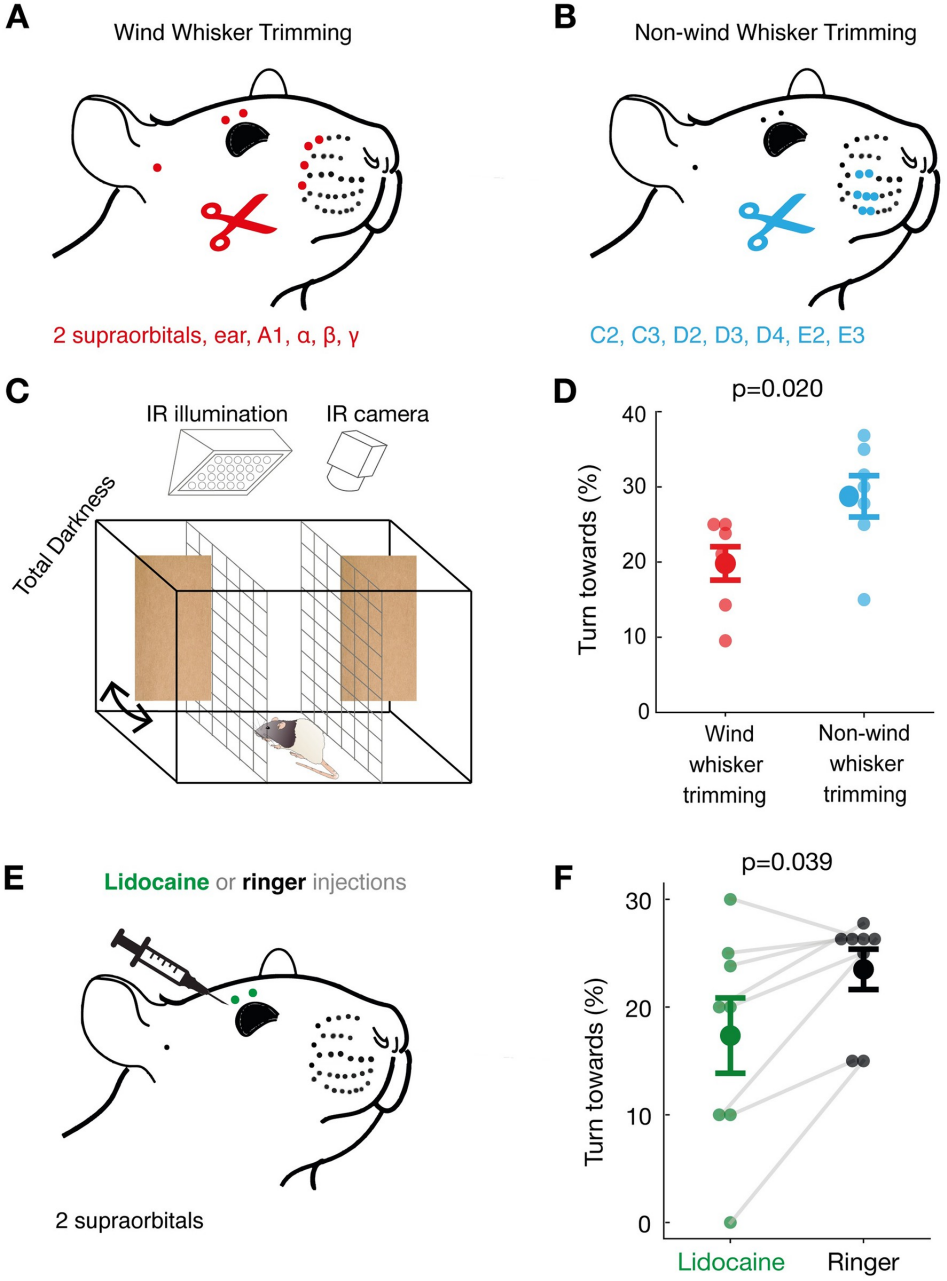
Differential effects of wind-whisker trimming and supra-orbital nerve blockade on rat airflow turning responses. **(A)** Wind-sensitive whiskers (2 supra-orbital, ear, A1, α, β, γ whiskers) were trimmed bilaterally in 7 rats. **(B)** Wind-insensitive whiskers (C2, C3, D2, D3, D4, E2, and E3) were trimmed bilaterally in another 7 rats. **(C)** Cardboard-flaps were used to deliver wind stimuli in the turning-behavior arena, as described in the Materials and methods section. **(D)** Wind-whisker-trimmed animals (red) turn towards flaps less strongly (*p* = 0.02, unpaired Mann–Whitney U-test, two-tailed, *N* = 7 animals) than non-wind-whisker-trimmed animals (blue). (**E)** The supra-orbital whisker follicles were targeted with lidocaine (green) or Ringer solution (gray) in 8 individuals in a paired procedure. **(F)** Lidocaine in supra-orbital whiskers (green) significantly decreased airflow turning responses relative to Ringer injection (*p* = 0.039; Wilcoxon signed-rank test, two-tailed, *N* = 8 animals, 20-21 trials each). All data underlying the figure can be accessed through https://figshare.com/s/07af3c1099b5b785acff.

Wind-responsive whiskers (2 supra-orbitals, ear, A1, *α*, *β*, and *γ* whiskers), as identified in our whisker tracking experiments, were trimmed in 7 rats (Fig 7A). A subset of wind-insensitive whiskers (C2, C3, D2, D3, D4, E2, and E3) were trimmed in 7 different rats, which had their wind-responsive whiskers intact (Fig 7B). Both sets of individuals were then submitted to cardboard-flap stimuli in complete darkness and were filmed (Fig 7C), as described in the previous section. Out of all trials, we counted each individual’s number of turns towards the stimulus. We found that on average, wind-whisker-trimmed individuals turned towards the stimulus 20% of the time, while non-wind-whisker-trimmed individuals turned towards the stimulus 29% of the time (*p* = 0.02, Fig 7D). Thus, removal of wind-responsive whiskers resulted in a stronger decrease in turning behavior than the removal of wind-insensitive whiskers.

We next asked if the supra-orbital whiskers alone play a role in wind-induced turning. To investigate this, we injected 8 individuals with either lidocaine or Ringer solution (as a negative control) locally at their supra-orbital whisker follicles and followed this with an injection of the respective other solution 24 h later (Fig 7E). After each injection, we subjected the animals to the cardboard-flap tests, as illustrated in Fig 5C. Therefore, we had 8 paired values for each condition. Seven out of 8 individuals showed a decrease in turning behavior for lidocaine when compared with Ringer solution (Fig 7F). The average turns towards the cardboard-flap stimulus were less frequent (18%) for lidocaine treatment than for Ringer treatment (23%, *p* = 0.039). We conclude that supra-orbital whiskers alone contribute significantly to airflow turning responses.

## Discussion

Here, we studied rat anemotaxis by combining whisker tracking, biomechanical analysis of whisker airflow responses, follicle analysis, somatosensory cortex recordings, behavioral analysis of airflow turning, and whisker interference by trimming and lidocaine injections. This diversity of methods led to a coherent pattern of results. Whiskers greatly differ in their airflow sensitivity and strongly wind-responsive whiskers—in particular the lSO—determine behavioral responses to airflow stimuli in rats.

### Differential sensitivity of rat whiskers and downstream cortices to airflow

Whisker tracking of large numbers of whiskers under a variety of airflow conditions suggested differential sensitivity of rat whiskers to airflow. The sheer amount of data acquired here reflects the power of a tracking software such as DeepLabCut [18,19] without which our analysis would not have been possible. The patterns of whisker airflow displacement were remarkably consistent across experiments. On one hand, we found that strong airflow displaces most whiskers. However, low airflow conditions lead to a differential engagement of whiskers, with only some (in particular, the supra-orbitals) showing strong movements. We controlled turbulent airflow to reach on average either 0.5 m/s (low) or 1.5 m/s (high) speeds, adding to other controlled airflow whisker displacement studies [6]. Our results on whiskers airflow-induced displacement indicates that only a narrow subset of whiskers are strongly engaged under low wind conditions (lSO, α, β, A1), being the lSO the one showing the largest displacements in most cases. Furthermore, lSO also exhibited the largest change across wind conditions (0.5 to 1.5 m/s), belonging to a larger subset of whiskers that displays substantial increases across conditions: lSO, sSO, α, β, δ, A1, A2, A3, B1, B2, B3, B4, and C1. Our biomechanical analysis enforced the idea of a differential whisker sensitivity to airflow. First, we found that strongly airflow responsive whiskers such as the supra-orbital have the highest whisker length-base diameter ratio. This property was directly correlated with wind-induced displacement. Second, even the extracted lSO whiskers show exceptionally strong airflow responses. Previous work in line with this indicates that the combination of longer length and a smaller whisker base diameter (i.e., high ratio) sets a series of resonance peaks for wind-sensitive whiskers that, starting from a lower frequency than other whiskers, enables mechanoreceptors at their base to transduce the wind-induced deflections more efficiently [8]. In other terms, the very low minimum mechanical resonance frequency of wind-sensitive whiskers [6] allows them to display a wider frequency range for mechanoreceptors to transduce wind vibrations [8]. This resulted in a large dynamic range.

The follicles of wind-sensitive whiskers differ from non-wind-sensitive whiskers by a more closed ring-wulst. Such ring-wulst differences are of great functional interest, because club-like endings on the ring-wulst are thought to form the most sensitive whisker afferents [21]. A synopsis of our observations points towards biomechanical specializations that endow the supra-orbital whiskers with strong omni-directional airflow sensing. In addition, given that wind speed is encoded as changes in the vibration plane of the whiskers [8] (i.e., parallel to perpendicular), whiskers with closest ring-wulsts, in the top-ocular corner of the rat’s face, may be more suited to transfer mechanical energy at low wind speeds. Together, the combination of wind-sensitive whiskers mechanical resonances [8] (which are tuned to the biophysical properties of their corresponding primary afferents) and the geometry of their ring-wulsts might empower them to accurately transduce airflow properties.

Cortical recordings confirmed—in direct comparison with the whisker pad region—that the supra-orbital region is particularly wind-sensitive and that its neurons convey larger amounts of information about wind stimuli. In accordance with this finding, it has been described that the responses of primary afferents at the trigeminal ganglion are strongly dependent on the whisker’s bending angle [8,25].

Our results suggest that even at speeds higher than the ones studied here, like those due to the rat’s self-motion, the differential airflow-engagement of both the whiskers and the cortical neurons will remain proportionally similar. In this sense, the top-ocular corner whiskers (lSO, α, β, and A1) and the neurons in the supra-orbital region displayed changes that given their described properties could easily be conserved over a broader range of wind speeds. Even if this is not the case, their ring-wulst aperture should always give these whiskers an extra advantage when sensing wind. It remains a matter of future research to determine whether and how the rat’s own movement would affect wind-related sensitivity.

### Rat anemotaxis

Previous work by Yu and colleagues [6] established the ability of rats to sense wind blown through tunnels. These abilities were diminished by trimming all facial whiskers. Our current work extends our knowledge of rat anemotaxic abilities. We demonstrate that rats show robust turning responses to both weak (hand-flaps) and strong (cardboard-flaps) airflow stimuli. Such turning responses confirm that rats can not only detect but also localize airflow stimuli. The task conditions (total darkness, no contact/little, or no audible sounds) and the diminished airflow responsiveness after whisker trimming or blockade clearly indicate that tactile stimuli induced anemotaxic turning. At least for the hand-flap, the evoked airflow currents—which the animals detect in distances of 10 cm or more—is small (measured airflow ≤0.3 m/s). Since a hand-flap is not categorically different from airflows induced by biologically relevant stimuli (such as a predator), we think such anemotaxic sensing might offer real-world advantages to nocturnal animals like rats. With exception of the fact that rats turn towards rather than away from hand-flap stimuli, our observations remind us of anemotaxic escape behaviors as they have been described in insects. Indeed, we wonder if the rat’s anemotaxic turning observed by us is also a defensive behavior that guards the animal against surprise attacks from the side or behind. The idea that supra-orbital, α, β, and A1 whiskers mediate defensive behaviors matches with their representation in the medial superior colliculus [26], where both visual [27] and electric stimulation [28] evoke defensive behaviors such as escape and freezing. In addition, it was shown by Saraf-Sinik and colleagues [29] that whisker-mediated perception can be modulated by the rat’s movement under changing environmental conditions (i.e., airflow), future research should be focused on the effect of motor variables in wind sensing, primarily in the top-ocular corner whiskers (lSO, α, β, and A1).

Independently of exact purpose and the underlying neural circuits, we find that anemotaxic turning is an extremely valuable behavioral assay for wind sensing in rats. As it requires no prior conditioning, the robustness of the behavior allowed us to screen wind-sensing abilities in large numbers (>20) of rats.

### The supra-orbital whiskers as wind antennae

The central conclusion from our work is that whiskers differ in their sensitivity to airflow stimuli. Specifically, the supra-orbital whiskers emerged as key sensors for wind stimuli from our analysis. These whiskers show maximal displacement to weak airflow stimuli, a response property that—according to *ex vivo* experiments—reflects the unique biomechanical properties of these whiskers. These properties align with a close ring-wulst and a high firing rate in response to wind stimuli. The very dorsal position and the upward bending very likely further enhances airflow sensitivity. At least in mice, supra-orbital whiskers appear to be actively whisked together with the mystacial whiskers [30]. The two supra-orbital whiskers are represented in two closely adjacent cortical barrels. Both whisker trimming and most of all the effects of lidocaine injections document the functional significance of supra-orbital whiskers for airflow sensing. The reduction of anemotaxic turning after supra-orbital lidocaine injections is a remarkable result, given that these bilateral injections targeted only 4 out of the roughly 300 rat whiskers.

Our data adds to the growing evidence that the functional diversity of whiskers enriches the rat’s sensory world [31,32]. The much-studied mystacial macrovibrissae seem to serve many functions, the microvibrissae mediate object contacts, trident whiskers engage in ground sensing and supra-orbital whiskers—according to several lines of evidence provided here—act as wind whiskers.

## Materials and methods

All experiments complied with regulations on animal welfare and were approved according to international law for animal welfare and approved by the State Office for Health and Social Affairs committee (LAGeSo) in Berlin (Animal license number: G0095-21 / 1.2), Woods Hole, USA (21-10C and 22-09E), and the Institutional Animal Care and Use Committee (IACUC) of the Fundación Instituto Leloir, Protocol No. 83 according to the Principles for Biomedical Research involving animals of the Council for International Organizations for Medical Sciences.

### Whisker displacement

Passive whisker movements were recorded in 5 anesthetized rats (P25–P32), and a total of 10 videos were analyzed. Acquisition was performed with a FLIR, ultra-HD camera at 100 frames per second (fps; Blackfly) under low-light conditions with led illumination of the facial whiskers. Each video lasted approximately 1 min. Airflow was directed frontally towards the face of the rats at low (0.5 m/s) and high (1.5 m/s) wind speed. The wind source was a computer fan (AITRIP, ECDG054) placed at 12.5 cm from the head. Wind speed was calibrated by taking measurements before each experiment (with a UNI-T UT363 BT anemometer), in front of the rats’ eyes and cheek, close to the whisker pad and following the same head’s coronal section to ensure wind flow was on average equivalent for all whiskers. Anemometer threshold = 0.3 m/s; sampling rate = 2 samples per sec.; resolution = 0.1 m/s.

Whisker video tracking was done using DeepLabCut (Mathis and colleagues (2018)) by defining a node at the whiskers end segment. Whisker displacement due to wind stimulation was calculated as the positive distance traveled by the whisker node in the 2D plane with respect to the median:

$$\sqrt{{\left(X-x\right)}^{2}+{\left(Y\;-\;y\right)}^{2}},$$

where X and Y are the median of position values for each 2D coordinate and x and y the observed position values. Whisker displacement and not speed was chosen given that they are informationally redundant as indicated by their Pearson correlation: values per rat 0.98, 0.95, 0.93, 0.97, and 0.92; *p*-value <10^−6^ in all cases.

### Micro-CT imaging

Whisker pads acquired from 7 male rats (P21–35) were scanned (5 to 6 follicles per whisker type were obtained). To achieve X-ray visibility of soft tissues, whole whisker pads were stained in 1% Lugol’s solution for 96 h or 1% phosphotungstic acid (PTA) for 7 days and single vibrissa follicles in 1% Lugol’s solution for 48 h, followed by washing in 0.1 M phosphate buffer (PB) for 1 to 4 h [33]. For fixation during scanning, samples were embedded in 2% to 4% agarose and placed in a 50 ml falcon tube (whisker pads) or a 1 μl pipette tip (single vibrissa follicle). Micro-CT scans were performed over a 360° rotation and images acquired every 0.2°, with exposure times between 1 to 2 s, with 40 to 60 kV and 70 to 100 μA with an YXLON FF20 CT system (YXLON International GmbH, Hamburg Germany) equipped with a Perkin Elmer Y Panel 4343 CT detector and 190 kV nano focus transmission tube. Helical scans allowed an effectively extended field of view in case of the whole whisker pad scans.

### Holotomography reconstructions

Micro-CT scans were reconstructed with the YXLON reconstruction software. Images were manually segmented in an extended version of the Amira software (AmiraZIBEdition 2022.17, Zuse Institute Berlin, Germany) and exported labels visualized with Dragonfly software (Dragonfly 2021.3, Object Research Systems (ORS), Montreal, Canada). Adobe Illustrator (Version 26.3.1) was used for the orientation and presentation of the data.

### Whisker morphology

Three to four whiskers per whisker type from 6 rats (P19–P25; male = 4, female = 2; this number includes the 4 male rats used in the micro-CT scans) were plucked to measure the whisker length and diameter. Representative whisker images from Fig 2 were taken either with an upright epifluorescence Zeiss microscope (Zen software, blue edition) with brightfield (5× objective, Zeiss) (Fig 2B, top panel) or using an AVT Pike f421b camera with a 60 mm Nikon macro lens (Measurement and Automation Explorer, National Instruments) (Fig 2B, bottom panel).

For length measurements, we used a Sony alpha 7s camera with an FE 2.8/90 Macro G OSS lens. For the whisker diameter, we used the images taken from the holotomography reconstructions. Whisker diameter was measured in a transverse section close to the ring sinus, once the thickness of the initial segment of the whisker reached a relatively constant thickness.

### Biomechanics

Two whiskers per whisker type from 3 rats (P19–P25; male = 2, female = 1) were plucked for the ex vivo assay (right side of the face). Whiskers were inserted by their base on clay in a linear array facing the same direction. Wind came mostly from the opposite direction of the resting curvature of the whiskers (see video 2). This was done to maximize whisker bending and to facilitate measurements, given that we observed the highest bending in this condition rather than when blowing wind in the same or perpendicular directions. To prevent wind from blowing directly towards the whiskers, we placed a plastic tube facing the whiskers 30 cm away from them with a fan placed on the distal end of the tube, away from the whiskers (the length of the tube was approximately 70 cm) and a loose paper towel on the proximal end of the tube, near the whiskers to attenuate wind intensity. The tube and the fan were approximately the same diameter (15 cm). Bending angle was reconstructed by superimposing 2 frames of a video where minimal and maximal deflection of the whisker was achieved. We used 75% of the total whisker length to trace a radius centered at the base of the whisker to calculate the bending angle. This procedure was repeated 6 times, once per whisker type. With this, we obtained 12 data points per whisker type. Images were acquired using a Logitech BRIO, ultra HD webcam (90 fps, Logitech).

### Cortical localization of supra-orbital whisker barrels

Long–Evans rats (P19–P25, *n* = 4) were anesthetized using urethane (1.4 g/kg i.p.). Incised tissue was locally anesthetized with lidocaine. A rectal probe monitored body temperature and a homeothermic blanket (FHC, Bowdoinham, Maine, USA) maintained it at 37 ± 0.5°C. For facial whisker barrel experiments, a craniotomy was made above the somatosensory cortex (3.5 mm posterior to bregma; 6.5 mm lateral to bregma). Broken glass electrodes filled with Ringer solution (NaCl 135, KCl 5.4, MgCl2 1, CaCl2 1.8, HEPES 5, in mM) were arranged to enter perpendicular to the cortex. Multi-unit activity was amplified using an Axoclamp 2B amplifier (Axon Instruments) and monitored (AM10 Grass Instruments) while moving in step coordinates centered around 6.3 mm posterior and 3.8 mm lateral to bregma and lightly moving the supra-orbital whiskers.

### Neuropixel recordings and wind stimulation

Male Long–Evans rats (*n* = 3) were kept in a temperature and humidity-controlled room with a 12 h:12 h light/dark cycle. Animals were allowed to have free access to clean food and water in standard rat cages. For surgery, animals were deeply anesthetized by applying intraperitoneal (ip) injections of urethane (1.5 g/kg body weight (BW)). The fur overlying the dorsal aspect of the animal skulls was shaved. Then, the rat was placed in a standard stereotaxic surgical apparatus (Narishige, Japan). The animal’s body temperature was measured with a rectal probe and kept at 36°C ± 0.5°C by a homeothermic blanket (FHC, Bowdoinham, Maine, USA). Before the surgical incision, the scalp of the animal was locally anesthetized by injecting 2% lidocaine solution. To access the barrel cortex, the skin was cut antero-posteriorly along the midline, and the remaining connective tissue on the skull was removed. The anchoring screws were inserted to the skull bone and a head-fixation post was then secured to these screws using UV-curable adhesive glue (Optibond; Altschul Dental, Mainz, Germany) and dental cement (Heraeus Kulzer, Hanau, Germany). Two Neuropixels probes were glued together (distance between the probes 2.0 to 2.2 mm), coated with lipophilic carbocyanine fluorescent dyes DiO or DiI, and lowered slowly into the barrel cortex. One of the probes targeted the supra-orbital whisker area at coordinates 3.8 mm posterior and 6.3 mm lateral, in a way that the second probe targeted the central whisker pad. Once the recording was stable, the supra-orbital and wind-insensitive whiskers were stimulated through mechanical and air puff means to confirm the position of both probes. If no clear response was observed, that is, if no supra-orbital and lower whisker pad response were observed on each of the Neuropixels, the probes were then moved until the expected supra-orbital/whisker pad response was found. Through this procedure, one of the probes showed responses exclusively during supra-orbital stimulation, while the second probe showed response exclusively for the wind-insensitive whiskers. Finally, a vent (AITRIP, ECDG054) was positioned in front of the animal at a distance of 12.5 cm and low (0.5 m/s) and high (1.5 m/s) wind stimuli was presented through a balanced randomized sequence of low, high and no-wind conditions (10 s each, 12 to 30 wind events per rat). Wind measurements were done using a UNI-T UT363 BT anemometer.

### Spike sorting

Spikes were detected from the high-pass filtered data using Kilosort 3.0 [34] and then the output clusters manually adjusted using the “phy” GUI (https://github.com/cortex-lab/phylab/phy). Clusters of neurons were assessed qualitatively in terms of their autocorrelogram (little presence of short-latency ISIs), spike amplitude and presence of a clear waveform modulation across channels. Neighboring clusters (up to 10 channels apart) were directly compared between each other in terms of cross-correlogram, waveform similarity per channel, and firing rate patterns (the latter, to avoid classifying as separate unit clusters that do not overlap in time). Clusters with high similarity index were also compared in the same manner. Only clusters satisfying all these criteria were considered in further analysis.

### Mutual information

Mutual information was calculated for each cell by computing the conditional probability, P(s|r) = P(s,r)/P(r), of the wind stimuli (S) given the cells firing rate (R) during each second relative to wind stimulation onset: −4 to 0 s as no wind (baseline) and from stimulus onset to 4 s with either low (0.5 m/s) or high (1.5 m/s) wind. Then, we determined the mutual information on each second after wind stimulus and on the baseline:

$$\sum_{R}\sum_{S}P\left(r\right)P\left(s|r\right){log}_{2}\left(\frac{P(s|r)}{P(s)}\right).$$

Mutual information during either low or high wind conditions was normalized by their respective baseline values.

### Response percentages

Histograms of the cell’s firing rate relative to wind stimulation (−4 to 4 s from wind onset; 1 s bins) were considered as independent variables of a generalized linear model using a Poisson distribution. Five categorical predictors corresponding to distinctive periods were included: baseline (−4 to 0 s) and seconds 1 to 4. Significance of activity in each post-stimulation second was assessed for each cell by comparing to baseline period. Accordingly, cells were classified in time as being excited or inhibited.

### Latency

Response latency was calculated using Baker and Gerstein (2001) [35] method (for a review, see Levakova and colleagues [36]). Cell’s firing rate was transformed into z-score and counts for each of 4 time bins (1 to 4 s after stimulus onset) were made if z-score surpassed a threshold (2 SD) and reached its maximum in that bin.

### Histochemical visualization of barrel patterns

The animals used for whisker mapping and Neuropixels recordings were deeply anesthetized and perfused transcardially with Ringer solution, followed by 4% paraformaldehyde (PFA). Brains were removed, hemispheres were separated, and cortices were flattened between 2 glass slides separated by clay spacers. Glass slides were weighed down with small ceramic weights for about 3 h. Afterwards, flattened cortices were stored overnight in 2% PFA and 80 μm sections were cut at room temperature on a vibratome. Sections were stained for cytochrome-oxidase activity using the protocol of Lauer and colleagues [37] and embedded in Mowiol mounting medium. Subsequently, barrel shapes were drawn with Neurolucida software (Microbrightfield, Colchester, Vermont, USA) using a Zeiss Axioplan microscope fitted with a 10× and 2× objective.

### Wind-sensing behavior

Long–Evans rats (P21–P32, male = 12; female = 13) were separated from littermates prior to behavioral testing. Behavioral videos were recorded (Basler acA1920, 100 fps) in a darkened room with the inner chamber covered with blackout curtains. The behavior box was illuminated with an infrared LED lamp. Two experimenters were positioned on opposing ends of the testing box and prepared for tests with hands or cardboard-flaps in position. Airflow measurements of hand and cardboard-flap stimuli were on average ≤0.3 m/s and 0.5 m/s, respectively. The testing animal was then placed in the center of the chamber, and a third experimenter cued the experimental flapper by name in a random sequence every 10 s, with a total of 13–20 trials per session.

Whisker trimming or lidocaine/Ringer injections were performed bilaterally in gently restrained animals under stereoscopic magnification and illumination within 10 min of behavior assessment. Injections were performed subcutaneously and directed to the area of origin for the supra-orbital whiskers. Wind-sensitive whiskers (2 supra-orbital, the ear, A1, α, β, and γ whiskers) or wind-insensitive whiskers (C2, C3, D2, D3, D4, E2, and E3) were trimmed with sharp scissors at the base of the skin without disturbing other whiskers. A day prior to the actual whisker trimming/lidocaine injections, the animals were habituated to the trimming/injection procedures in sham trimming/injection procedures in order to minimize stress on the day of the actual experiment. In such sham procedures, animals were gently restrained, positioned under the microscope and a pair of scissors was brought close to the animal’s face.

### Statistics

Most of our dataset did not satisfy normality criteria, so we applied nonparametric statistics. We analyzed data from binomial distributions with χ^2^ and Fisher’s exact test. Mann–Whitney, Wilcoxon, or Kruskal–Wallis tests were employed to analyze 2 unpaired groups, 2 paired groups, or more than 2 unpaired groups, respectively. Post hoc analysis was carried out using Tukey (Fig 1 and S2, S3 and S4 Figs) or Dunn’s test (Fig 2F). Data was expressed as the median ± interquartile range (IQR) or the mean ± the standard error of the mean (SEM). We only report differences which were significant and relevant to the experiment. In all cases, *p* < 0.05 was the statistical threshold. The analyses were done using Python 3.7 or MATLAB (MathWorks, Natick, Massachusetts, USA).

### Shuffling statistics of whisker parameters

Chance-level statistics were constructed to determine an optimal arrangement for the whisker length-diameter ratio and ring-wulst aperture along the whole supra-orbital-whisker pad region (Figs 2D and 3F, respectively). The arrangement with the least mean variance was considered as the optimal and employed as grouping criteria for further analysis.

Six possible arrangements were considered: arcs, rows, semicircles (from A1), oblique 45° (from A1), oblique 135° (from A4), and opposite semicircle (from E4). We first calculated the variance inside each arrangement group (e.g., inside each semicircle) and took the mean across them as an estimate of the variance of the whole arrangement. A *p*-value for that estimation was then calculated by constructing a shuffle distribution of the mean variance for that arrangement. To this aim, data points position on the pad were randomized and the mean variance calculated for that arrangement. This procedure was repeated 10,000 times to create the shuffle distribution. Note that for both variables, the semicircular arrangement exhibited the least mean variance when comparing the observed value against the shuffle distribution for that arrangement.

## Supporting information

S1 Movie Whisker movements in low (0.5 m/s) airflow conditions. Note the selective engagement of supra-orbital whiskers in low airflow conditions. https://figshare.com/s/5d9b0586c062bc5d374c.
(MP4)

S2 Movie Whisker movements in high (1.5 m/s) airflow conditions. https://figshare.com/s/84bd74eb6c09f1eb055d.
(MP4)

S3 Movie Airflow at low (0.5 m/s) airflow conditions. https://figshare.com/s/a4f3621d9f136fcc0973.
(MP4)

S4 Movie Airflow at high (1.5 m/s) airflow conditions. https://figshare.com/s/326a94986bced4b298fd.
(MP4)

S5 Movie Airflow whisker responses recorded ex vivo with extracted whiskers. https://figshare.com/s/9c9c2aca5f87ecab31b1.
(MP4)

S1 Fig Wind flow characterization. **(A)** Example traces of the wind speed for low (blue; 0.5 m/s) and high (red; 1.5 m/s) wind conditions during a 10 s window. Arrows indicate sudden changes in the speed, indicative of turbulence. **(B)** Representative images of wind flow in the low (top) and high (bottom) wind conditions. Wind flow was visible by placing smoking dry ice behind the computer fan in dim light. Yellow dashed line indicates the point where the rats heads were placed during the experiments (see Figs 1 and 5). **(C)** As in A but for the average of four sweeps per wind condition. Wind speed takes 1.5 s and 2.5 s to surpass 80% (solid) and 95% (dashed) of the mean value, respectively. **(D)** Wind speed distribution for the low (left) and high (right) wind conditions. All data underlying the figure can be accessed through https://figshare.com/s/969b169e474aa1a4267d.
(TIF)

S2 Fig Differential displacement of whiskers responses to low wind (0.5 m/s) in each rat. Bar plots depicting whiskers displacement (mean ± SEM) as in Fig 1C, for 4 other rats. Rat 2: H (25, 152,411) = 77,052.98, *p* < 0.0001; lSO displaced significantly more than all other whiskers but α. Rat 3: H (25, 180,257) = 82,480, *p* < 0.0001; lSO displaced significantly more than all other whiskers but β. Rat 4: H (25, 219,309) = 117,402, *p* < 0.0001; lSO displaced significantly more than all other whiskers but A1 and α. Rat 5: H (25, 200,640) = 81,418, *p* < 0.0001; lSO displaced significantly more than all other whiskers. All data underlying the figure can be accessed through https://figshare.com/s/a004130cea2a039bc598.
(TIF)

S3 Fig Displacement ratio for all analyzed whiskers and its relation to wind speed. **(A)** Distribution of whiskers displacement ratio (high/low wind) by whisker type. Each circle represents the value belonging to 1 rat and bars represent mean ± SEM. **(B)** Same ratio as in A but for the median value of each distribution. Wilcoxon test between displacement ratios and 1: *p* = 0.01. Note that nearly a dozen values are far from 1, probably contributing the most to the overall effect. These values belong to whiskers lSO, sSO, α, β, δ, A1, A2, A3, B1, B2, B3, B4, and C1. All data underlying the figure can be accessed through https://figshare.com/s/83ea240cbed0b729a6d5.
(TIF)

S4 Fig Whisker biomechanics in relation to whisker displacement. **(A)** Boxplot for the whisker length-base diameter ratio normalized by the mean lSO ratio. Ratios were arranged according to the semicircular configuration, which exhibited the lowest observed *p*-value with respect to a shuffled distribution for that configuration (semicircular, *p*-value = 0.018). Kruskal–Wallis test, semicircular grouping as factor [H (6, 69) = 24.07, *p* = 0.0005]. Tukey post hoc indicated that groups SC2, 3 and 4 differed significantly from lSO (*p* < 0.04). Additionally, group SC1 differed from SC4 (*p* = 0.001). **(B)** Pearson correlation between whisker length and whisker base diameter with whiskers displacement. Rho and *p*-value indicated. All data underlying the figure can be accessed through https://figshare.com/s/9926eea051fcc1a613b0.
(TIF)

S5 Fig Whisker biomechanics in relation to ring-wulst aperture. **(A)** Boxplot for the ring-wulst aperture normalized by the mean lSO aperture. Apertures were arranged according to a semicircular configuration, which exhibited the lowest observed *p*-value with respect to a shuffled distribution for that configuration (semicircular, *p*-value <0.0001). Kruskal–Wallis test, semicircular grouping as factor [H (6, 122) = 61.69, *p* < 0.0001]. Tukey post hoc indicated that groups SC2, 3 and 4 differed significantly from lSO (*p* < 0.02). In addition, SSO and SC1 differed from SC3 and 4 (*p* < 0.04) and A1 from SC4 (*p* = 0.02). Finally, SC2 differed from SC 4 (*p* = 0.03). **(B)** From left to right, Pearson correlations between whisker length, whisker base diameter and the ratio between them against ring-wulst aperture. lSO whisker indicated (red). Rho and *p*-value indicated. All data underlying the figure can be accessed through https://figshare.com/s/be75cb161f8b9eca5bb3.
(TIF)

S6 Fig Response latency and mutual information for barrel cortex neuronal activity after different wind stimulations. **(A)** Histograms for the number of cells reaching their maximum response after stimulation onset (1 to 4 s). Latency was calculated using firing rate z-score and a classification threshold (2 SD; Levakova and colleagues). **(B)** Mutual information of firing rate given a stimulus (low wind, 0.5 m/s, blue; high wind, 1.5 m/s, red), normalized by the average during the 4 s previous to stimulation. Comparison of normalized information against 1 (two-tailed Wilcoxon test). Only values belonging to the supra-orbital region were significant. Second 1: (low wind, *p* = 0.001; high wind, *p* = 0.002); Second 2: (low wind, *p* = 0.0007; high wind, *p* < 0.0001); Second 3: (low wind, *p* = 0.001). Bonferroni’s correction α = 0.003. All data underlying the figure can be accessed through https://figshare.com/s/2c51be09f1a3d6d295bb.
(TIF)

## Abbreviations

BW: body weight
IQR: interquartile range
lSO: long supra-orbital
micro-CT: micro computed tomography
PSTH: peristimulus time histogram
PTA: phosphotungstic acid
sSO: short supra-orbital
