# Supplementary figures and images for: Supra-orbital whiskers act as wind-sensing antennae in rats
Source: PLoS Biol. 2023 Jul 6;21(7):e3002168. doi: 10.1371/journal.pbio.3002168 (PMC10325054; doi:10.1371/journal.pbio.3002168)

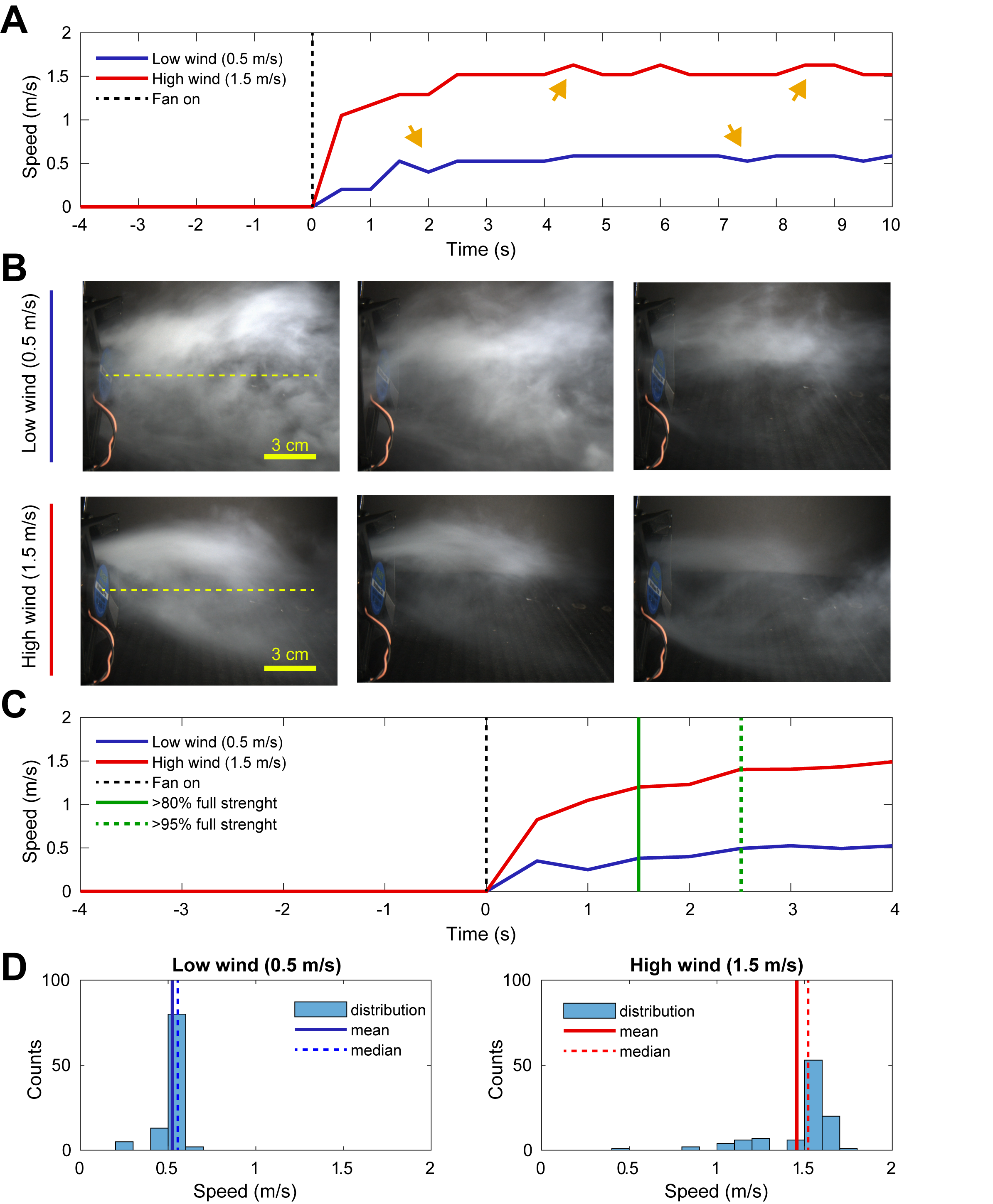

Supplement: S1 Fig — (A) Example traces of the wind speed for low (blue; 0.5 m/s) and high (red; 1.5 m/s) wind conditions during a 10 s window. Arrows indicate sudden changes in the speed, indicative of turbulence. (B) Representative images of wind flow in the low (top) and high (bottom) wind conditions. Wind flow was visible by placing smoking dry ice behind the computer fan in dim light. Yellow dashed line indicates the point where the rats heads were placed during the experiments (see Figs 1 and 5). (C) As in A but for the average of four sweeps per wind condition. Wind speed takes 1.5 s and 2.5 s to surpass 80% (solid) and 95% (dashed) of the mean value, respectively. (D) Wind speed distribution for the low (left) and high (right) wind conditions. All data underlying the figure can be accessed through https://figshare.com/s/969b169e474aa1a4267d. (TIF) [file pbio.3002168.s006.tif]

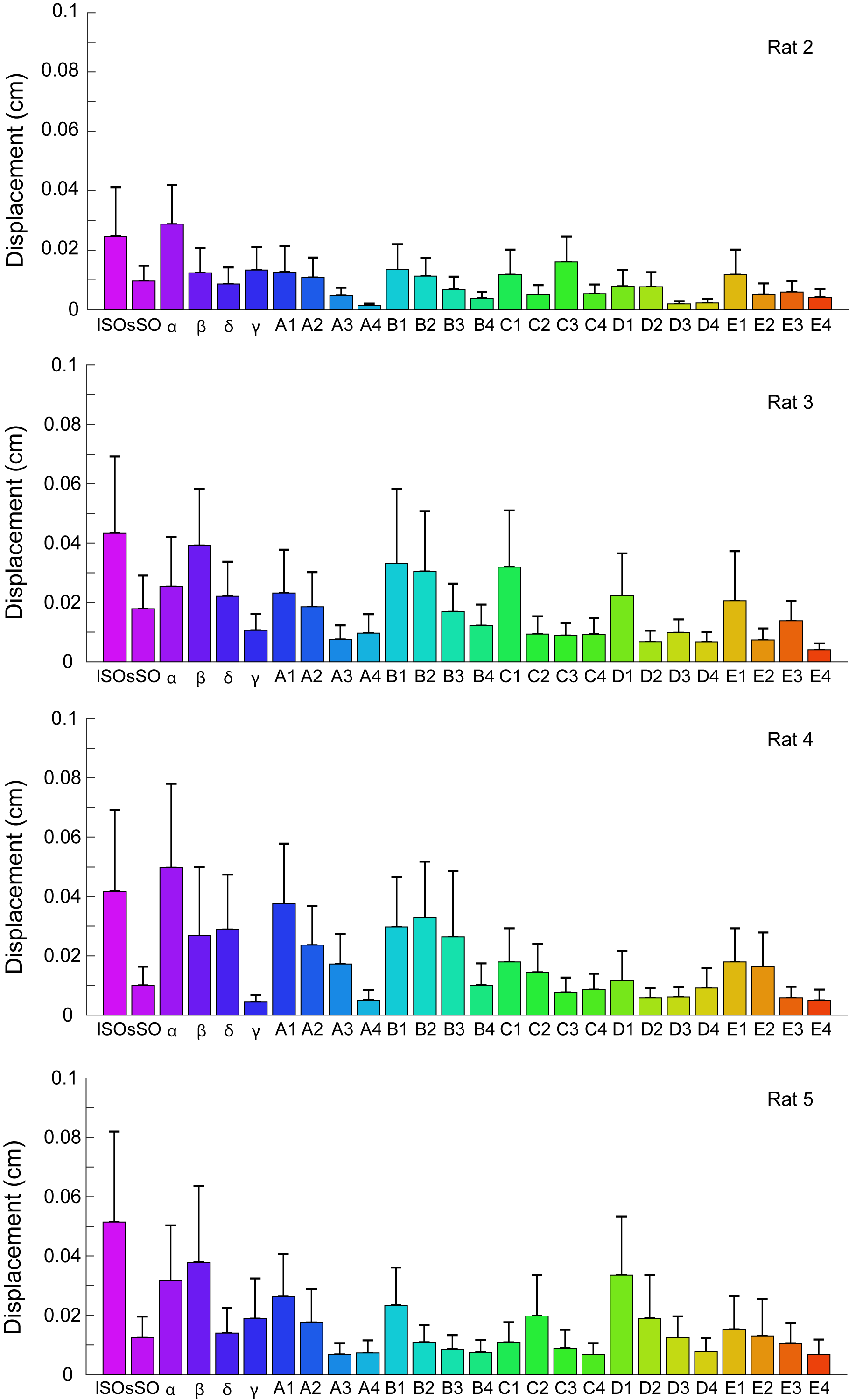

Supplement: S2 Fig — Bar plots depicting whiskers displacement (mean ± SEM) as in Fig 1C, for 4 other rats. Rat 2: H (25, 152,411) = 77,052.98, p < 0.0001; lSO displaced significantly more than all other whiskers but α. Rat 3: H (25, 180,257) = 82,480, p < 0.0001; lSO displaced significantly more than all other whiskers but β. Rat 4: H (25, 219,309) = 117,402, p < 0.0001; lSO displaced significantly more than all other whiskers but A1 and α. Rat 5: H (25, 200,640) = 81,418, p < 0.0001; lSO displaced significantly more than all other whiskers. All data underlying the figure can be accessed through https://figshare.com/s/a004130cea2a039bc598. (TIF) [file pbio.3002168.s007.tif]

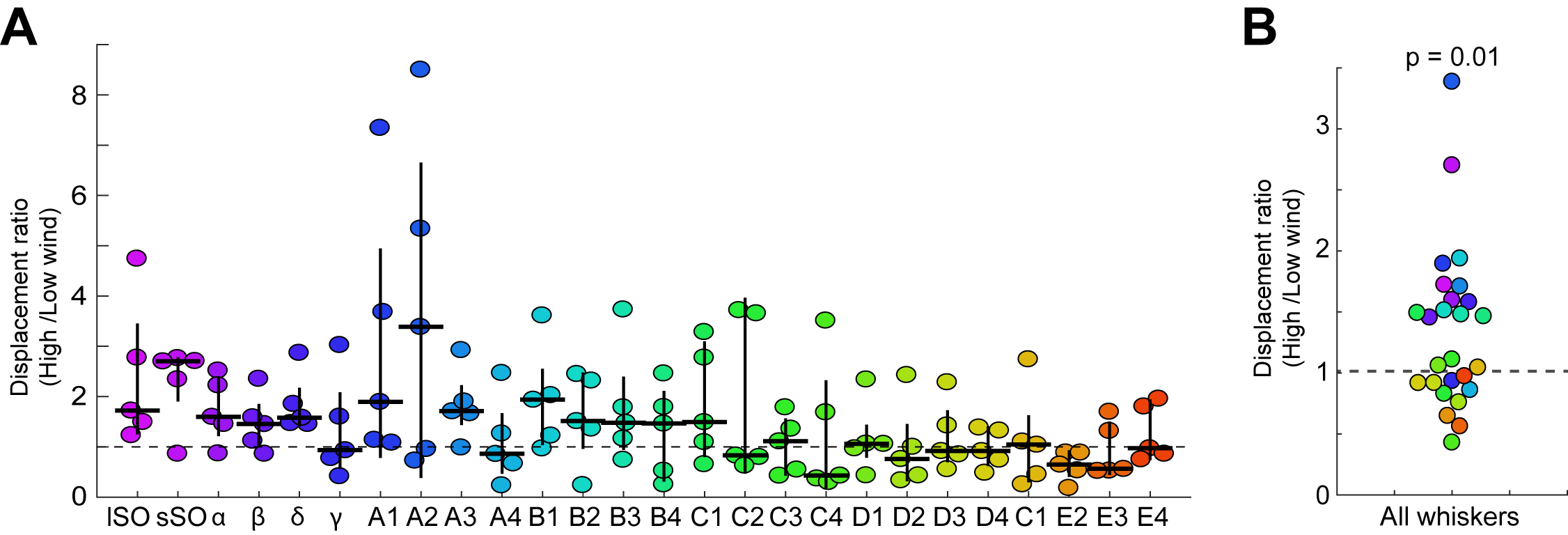

Supplement: S3 Fig — (A) Distribution of whiskers displacement ratio (high/low wind) by whisker type. Each circle represents the value belonging to 1 rat and bars represent mean ± SEM. (B) Same ratio as in A but for the median value of each distribution. Wilcoxon test between displacement ratios and 1: p = 0.01. Note that nearly a dozen values are far from 1, probably contributing the most to the overall effect. These values belong to whiskers lSO, sSO, α, β, δ, A1, A2, A3, B1, B2, B3, B4, and C1. All data underlying the figure can be accessed through https://figshare.com/s/83ea240cbed0b729a6d5. (TIF) [file pbio.3002168.s008.tif]

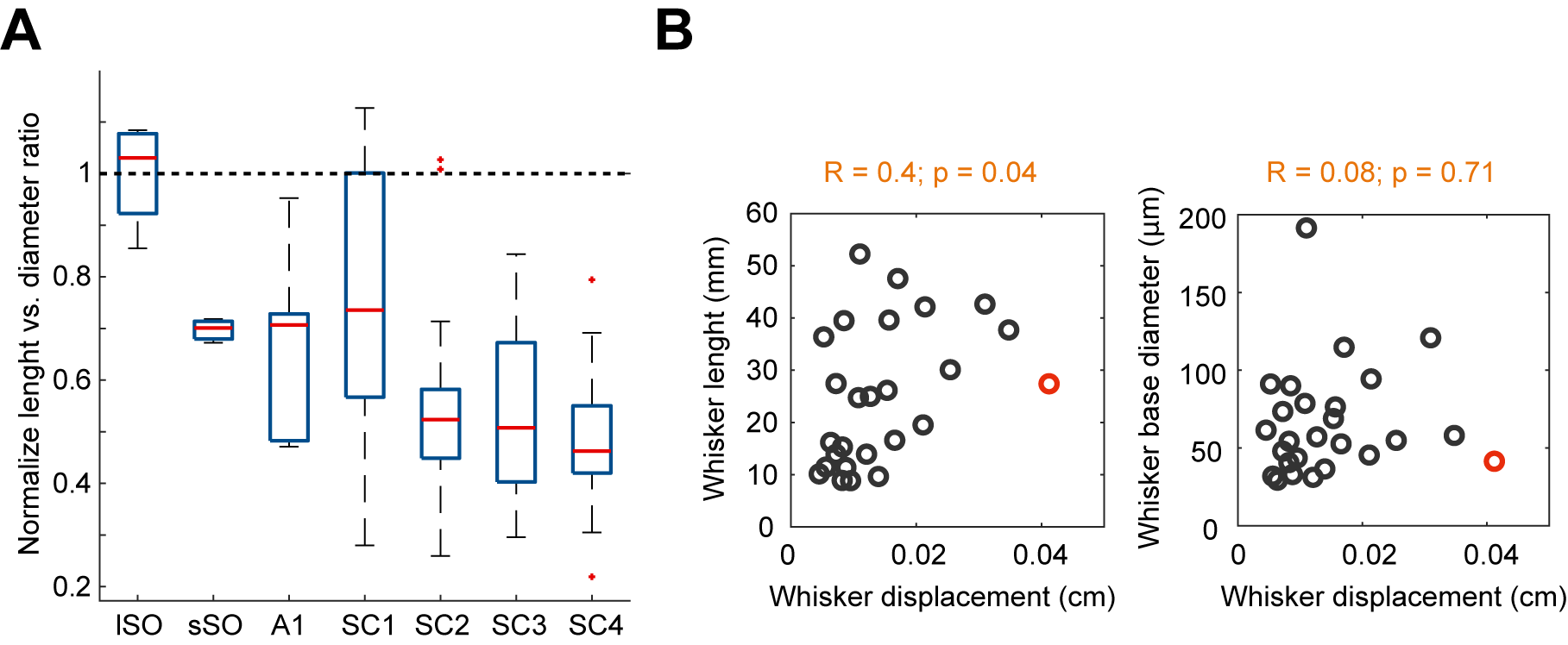

Supplement: S4 Fig — (A) Boxplot for the whisker length-base diameter ratio normalized by the mean lSO ratio. Ratios were arranged according to the semicircular configuration, which exhibited the lowest observed p-value with respect to a shuffled distribution for that configuration (semicircular, p-value = 0.018). Kruskal–Wallis test, semicircular grouping as factor [H (6, 69) = 24.07, p = 0.0005]. Tukey post hoc indicated that groups SC2, 3 and 4 differed significantly from lSO (p < 0.04). Additionally, group SC1 differed from SC4 (p = 0.001). (B) Pearson correlation between whisker length and whisker base diameter with whiskers displacement. Rho and p-value indicated. All data underlying the figure can be accessed through https://figshare.com/s/9926eea051fcc1a613b0. (TIF) [file pbio.3002168.s009.tif]

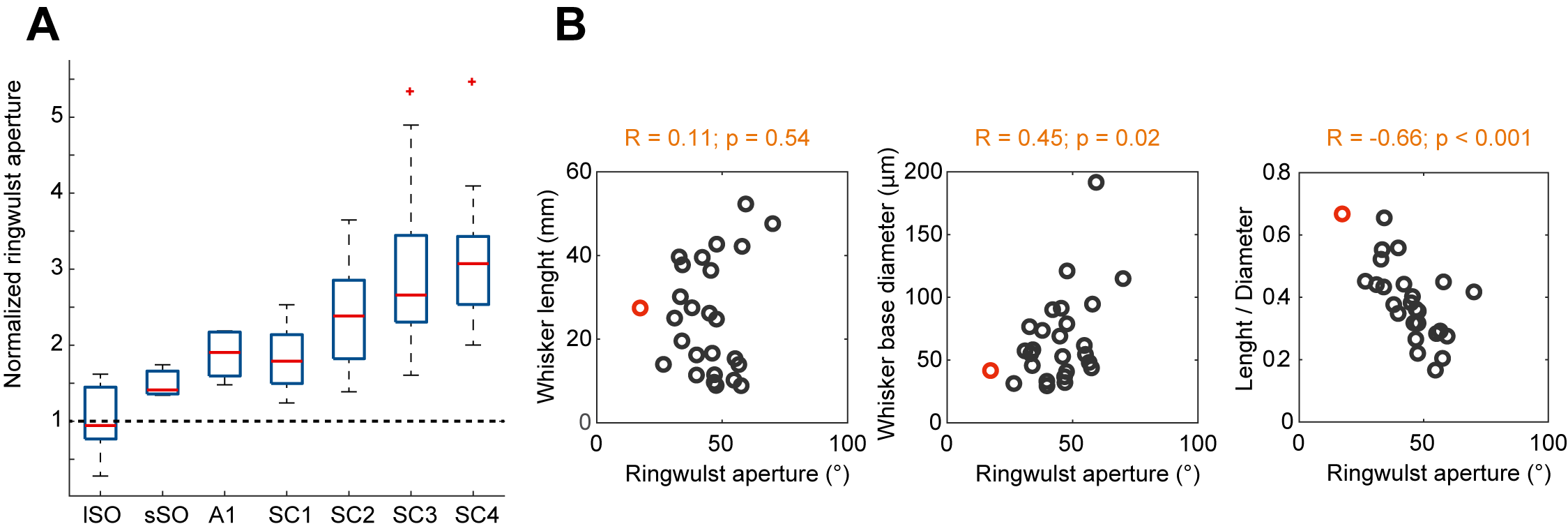

Supplement: S5 Fig — (A) Boxplot for the ring-wulst aperture normalized by the mean lSO aperture. Apertures were arranged according to a semicircular configuration, which exhibited the lowest observed p-value with respect to a shuffled distribution for that configuration (semicircular, p-value <0.0001). Kruskal–Wallis test, semicircular grouping as factor [H (6, 122) = 61.69, p < 0.0001]. Tukey post hoc indicated that groups SC2, 3 and 4 differed significantly from lSO (p < 0.02). In addition, SSO and SC1 differed from SC3 and 4 (p < 0.04) and A1 from SC4 (p = 0.02). Finally, SC2 differed from SC 4 (p = 0.03). (B) From left to right, Pearson correlations between whisker length, whisker base diameter and the ratio between them against ring-wulst aperture. lSO whisker indicated (red). Rho and p-value indicated. All data underlying the figure can be accessed through https://figshare.com/s/be75cb161f8b9eca5bb3. (TIF) [file pbio.3002168.s010.tif]

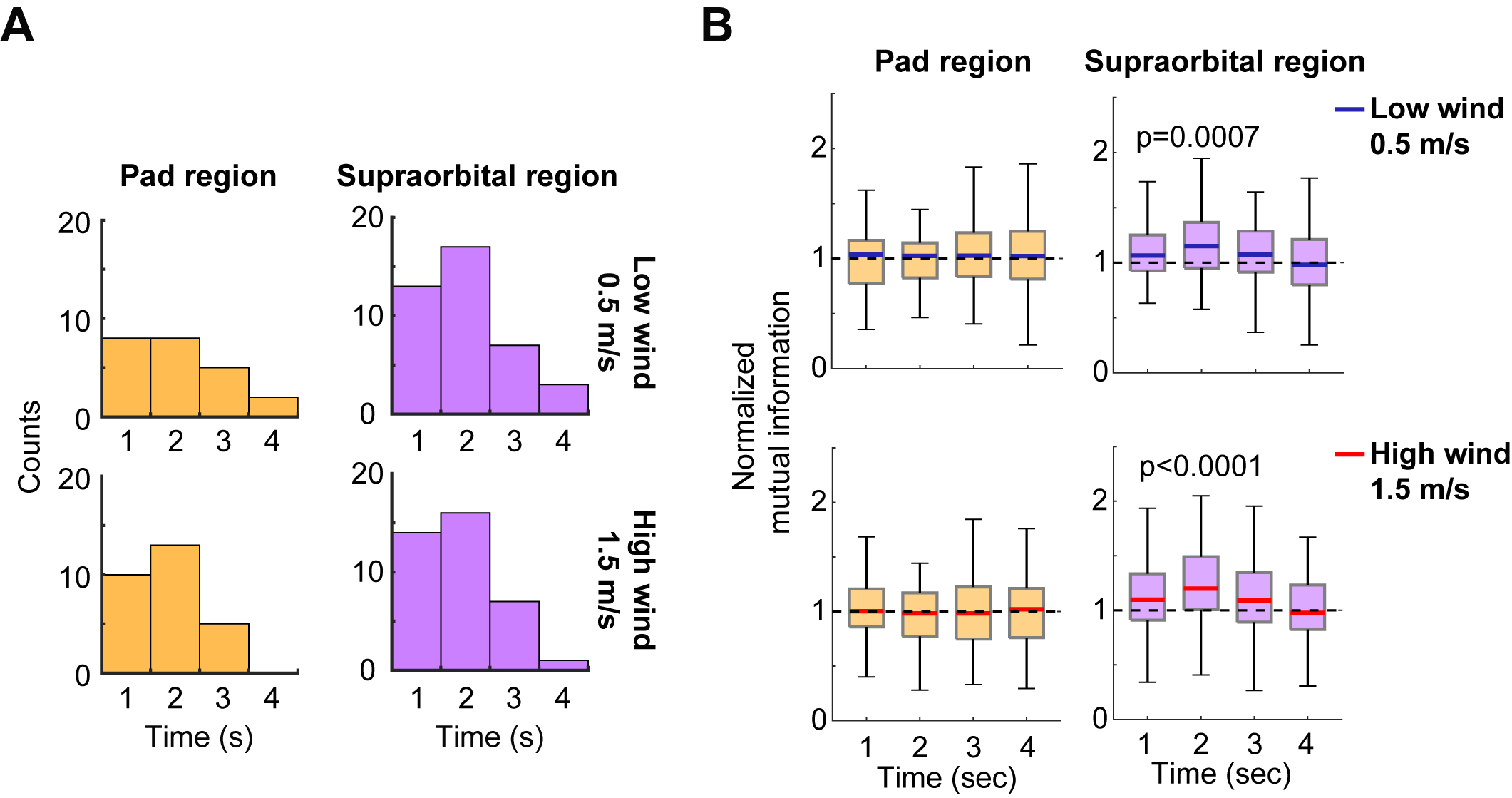

Supplement: S6 Fig — (A) Histograms for the number of cells reaching their maximum response after stimulation onset (1 to 4 s). Latency was calculated using firing rate z-score and a classification threshold (2 SD; Levakova and colleagues). (B) Mutual information of firing rate given a stimulus (low wind, 0.5 m/s, blue; high wind, 1.5 m/s, red), normalized by the average during the 4 s previous to stimulation. Comparison of normalized information against 1 (two-tailed Wilcoxon test). Only values belonging to the supra-orbital region were significant. Second 1: (low wind, p = 0.001; high wind, p = 0.002); Second 2: (low wind, p = 0.0007; high wind, p < 0.0001); Second 3: (low wind, p = 0.001). Bonferroni’s correction α = 0.003. All data underlying the figure can be accessed through https://figshare.com/s/2c51be09f1a3d6d295bb. (TIF) [file pbio.3002168.s011.tif]
